# Computing the origin and evolution of the ribosome from its structure — Uncovering processes of macromolecular accretion benefiting synthetic biology

**DOI:** 10.1016/j.csbj.2015.07.003

**Published:** 2015-07-26

**Authors:** Gustavo Caetano-Anollés, Derek Caetano-Anollés

**Affiliations:** a Evolutionary Bioinformatics Laboratory, Department of Crop Sciences, University of Illinois at Urbana-Champaign, 1101W. Peabody Drive, Urbana, IL 61801, USA; b C.R. Woese Institute for Genomic Biology, University of Illinois, Urbana, IL 61801, USA

**Keywords:** Molecular structure, Origin of life, Phylogenetic analysis, rRNA, Ribosomal evolution, Translation, Proteome, Protein structural domains, Molecular functions, Evolutionary genomics

## Abstract

Accretion occurs pervasively in nature at widely different timeframes. The process also manifests in the evolution of macromolecules. Here we review recent computational and structural biology studies of evolutionary accretion that make use of the ideographic (historical, retrodictive) and nomothetic (universal, predictive) scientific frameworks. Computational studies uncover explicit timelines of accretion of structural parts in molecular repertoires and molecules. Phylogenetic trees of protein structural domains and proteomes and their molecular functions were built from a genomic census of millions of encoded proteins and associated terminal Gene Ontology terms. Trees reveal a ‘metabolic-first’ origin of proteins, the late development of translation, and a patchwork distribution of proteins in biological networks mediated by molecular recruitment. Similarly, the natural history of ancient RNA molecules inferred from trees of molecular substructures built from a census of molecular features shows patchwork-like accretion patterns. Ideographic analyses of ribosomal history uncover the early appearance of structures supporting mRNA decoding and tRNA translocation, the coevolution of ribosomal proteins and RNA, and a first evolutionary transition that brings ribosomal subunits together into a processive protein biosynthetic complex. Nomothetic structural biology studies of tertiary interactions and ancient insertions in rRNA complement these findings, once concentric layering assumptions are removed. Patterns of coaxial helical stacking reveal a frustrated dynamics of outward and inward ribosomal growth possibly mediated by structural grafting. The early rise of the ribosomal ‘turnstile’ suggests an evolutionary transition in natural biological computation. Results make explicit the need to understand processes of molecular growth and information transfer of macromolecules.

## 1 Introduction

“As we trace the changes in structure or function back through time, we must bear in mind that all of the structures and functions of the cell may be simpler. We are then dealing with primitive components ancestral to those seen today.” Eck and Dayhoff [1]

Galaxies evolve by accretion, gravitational interactions, harassment, and dry and wet mergers of stars, gas and dust clouds [2]. Stars form by gravitational collapse within giant molecular clouds and accrete circumstellar disks of orbiting matter that spiral inward towards the growing central bodies [3]. Planets arise from the proto-planetary disks of gas and solids by a process of accretion and N-body interactions [4]. Unsurprisingly and at the other end of the spectrum, macromolecules in the biological world arise and evolve by similar processes of accretion, adding component parts to growing molecules, which also interact and merge with other molecular bodies to form molecular complexes and repertoires and higher order molecular and cellular structure.

The dynamics of macromolecular accretion involves a number of agents of genetic change, including point mutations, insertions, deletions, rearrangements, fusions, and fissions, and a multiplicity of interactions that prompt nucleating foci for growing molecules. While some of these processes materialize relatively quickly in lineages of organisms others take millions to billions of years to unfold. Their rates and distributions are not well understood. The protein world for example is incredibly vast when studied at polymer sequence level but its diversity can be currently summarized with only ~ 1200 fold designs that distil the fundamental topologies of their atomic 3-dimensional (3D) structures [5], [6]. Amino acid sequences showcase a limited alphabet that changes constantly by mutation. Sequences become saturated with recent mutations. They are poor repositories of historically deep phylogenetic information. In contrast, the structure of proteins and nucleic acids is highly conserved at the evolutionary level and carries considerable phylogenetic information [5]. As advanced by Epstein [7], molecular structures were initially recognized as being more refractory to the effects of mutation than nucleotide or protein sequences [1]. This results in a very limited repertoire of fold designs, a fact that was already evident in the crystallographic entries of the seventies [8], [9]. This is now made evident by man-made data-mining structural classifications. Thus, information in protein structure persists longer than in primary sequence and can be suitably mined with phylogenetic methods [10]. Since the history of individual protein or nucleic acid macromolecules can span millions to billions of years, it is therefore appropriate to study macromolecular change with highly conserved structural features [5].

Here we review studies that focus on the nucleation of biological structure into ‘modules’, their diversification and gradual accumulation, and their accretion to form high-level structures. The definition of modular parts is entirely dependent on the embedding *dynamical system* (see glossary in Table 1) [11]. Modules are sets of integrated parts that cooperate to perform a task and interact more extensively with each other than with other parts and modules of the system [12]. Modules can emerge through a biphasic process of diversification [13]. In the first phase, parts are at first weakly linked and associate variously. As they diversify, parts compete with each other and are selected for performance. The emerging interactions constrain their structure and associations. This causes parts to self organize into modules with tight linkage. In the second phase, variants of the modules evolve and become new parts for a new generative cycle of higher-level organization.

We explore evidence supporting macromolecular accretion as an evolutionary process tailoring the generation of biological modules and complexity. We start by discussing accretion of parts in molecular repertoires, focusing on the collective of all proteins of an organism, the proteome. We then turn to accretion of substructures in RNA molecules. Finally, we use the ribosome as an example of molecular accretion and tight coevolution of both protein and RNA modular parts. We stress the need to uncover processes of molecular growth and constraints responsible for molecular structure that can benefit the enterprise of synthetic biology.

## 2 An initial note on macromolecular history and scientific inquiry

The neo-Kantian philosopher of science Wilhelm Windelband proposed the existence of two general conceptual frameworks of scientific inquiry, the *ideographic* and the *nomothetic* methods [14]. As we will show, both of them can be used to study biological phenomena and to uncover in our case transformation processes in macromolecules. The ideographic framework that is typical of phylogenetic analysis surveys present-day molecular structures and builds *phylogenies* (Table 1) to make *retrodictive* statements about the past. It is historical. In turn, the nomothetic framework proposes *universal* statements about these same structures, which serve to make predictions about structural change. The nomothetic framework makes inferences without invoking history and is ultimately statistical.

The field of cladistics embodies the essence of ideographic thinking [15]. It makes explicit the exploration of ‘discovery operations’ necessary to study biological phenomena that vary extensively across time and space. Discovery operations are “sets of decision rules used to generate and choose among competing empirical claims” [15]: rules select empirical tests capable of decisive falsification of competing explanatory hypotheses. According to Willi Hennig and his school, ‘shared and derived’ features useful for retrodiction (termed phylogenetic *characters*; Table 1) provide evolutionary evidence [16]. These so called ‘synapomorphies’ represent in themselves discovery operations that determine the relative ancestrality of alternative character states as they induce directionality (polarity) of change (*character transformation*) (Table 1). This is a crucial part of the evolutionary model, which in turn is part of the trilogy of observations (data), parametric or non-parametric model of change, and phylogenetic tree or network representation of change (see [17] for a view of competing discovery operations). The historical framework of biology is supported by an ensemble of primary axioms of the highest level of universality: (1) evolution occurs, with change entailing spatiotemporal continuity (Leibnitz's principle of continuity), (2) only one phylogeny of extant and extinct biological entities (organisms and their component parts) exists as a consequence of descent with modification, and (3) characters are transmitted via genealogical descent [18]. These axioms must be fulfilled even when evolution proceeds in reticulate manner or when changes are saltatory, as long as information of axiom 3 is preserved by genetic or compositional codes. The explanatory power of the historical framework derived from phylogenies and timelines is strongly grounded in “reciprocal illumination” [16], a principle that evaluates how each ‘primary homology’ statement of character evolution (e.g. phylogenetically informative structural features of molecules, gene content) agrees with the overall favored evolutionary hypothesis obtained from all available data (e.g. [19])(Table 1). Agreements reformulate homology hypotheses iteratively to maximize explanatory power through test and corroboration [20]. Thus, phylogenetic analysis often operates under the Popperian pillars of content of theories and degree of corroboration.

In sharp contrast, chemistry and biochemistry are typical nomothetic sciences searching for general laws that can explain the chemical complexity of the inanimate and living worlds. The ultimate goal of these falsely perceived ‘end-goal’ disciplines is to uncover first principles governing the essence and behavior of atoms and molecules. While there is currently a need of a ‘pan-ideographic’ unification that integrates biology, chemistry and physics [21], the field of evolutionary bioinformatics now makes the challenge of choosing scientific frameworks explicit, especially when attempting to explain the origin and evolution of molecular structure and the emergence of biological complexity.

## 3 Accretion of macromolecular repertoires in proteome evolution

Protein structures are organized in a nested hierarchy of structural modules, which appear recurrently in different molecular contexts at both protein and proteome levels [5]. For example, elements of secondary structure of proteins (helices, strands, turns and loops) can result from a *frustrated dynamics* (Table 1) of interactions of the polypeptide backbone with itself and the surrounding molecular environment when molecules transition from disorder to structural order in the process of folding [22]. While frustrated backbone energetics is negotiated, the energetics of side-chain interactions ensures that low energy conformations of individual residues are achieved and that the entire structural core is stabilized by formation of compact and well-packed structures, largely mediated by van der Waals forces and hydrophobic collapse. These folded cores are the structural domains of proteins, 3D atomic arrangements of elements of secondary structure that fold into well-packed structural units [23], [24] and are evolutionarily conserved [25], [26], [27]. Domains fold and function largely independently [28]. They establish a multiplicity of intramolecular interactions, which contribute to the overall stability of the molecules [29]. Not all proteins however fold into individual cores when prompted by the nucleation process. Multidomain proteins, which globally make a significant minority (26–32%) of proteins in proteomes (they are highly represented in eukaryotes), sometimes contain a multiplicity of domains that on average are substantially smaller than domains in single domain proteins [30]. Finally single domain and multidomain proteins form open and close homomers [31].

**Fig. 1:**
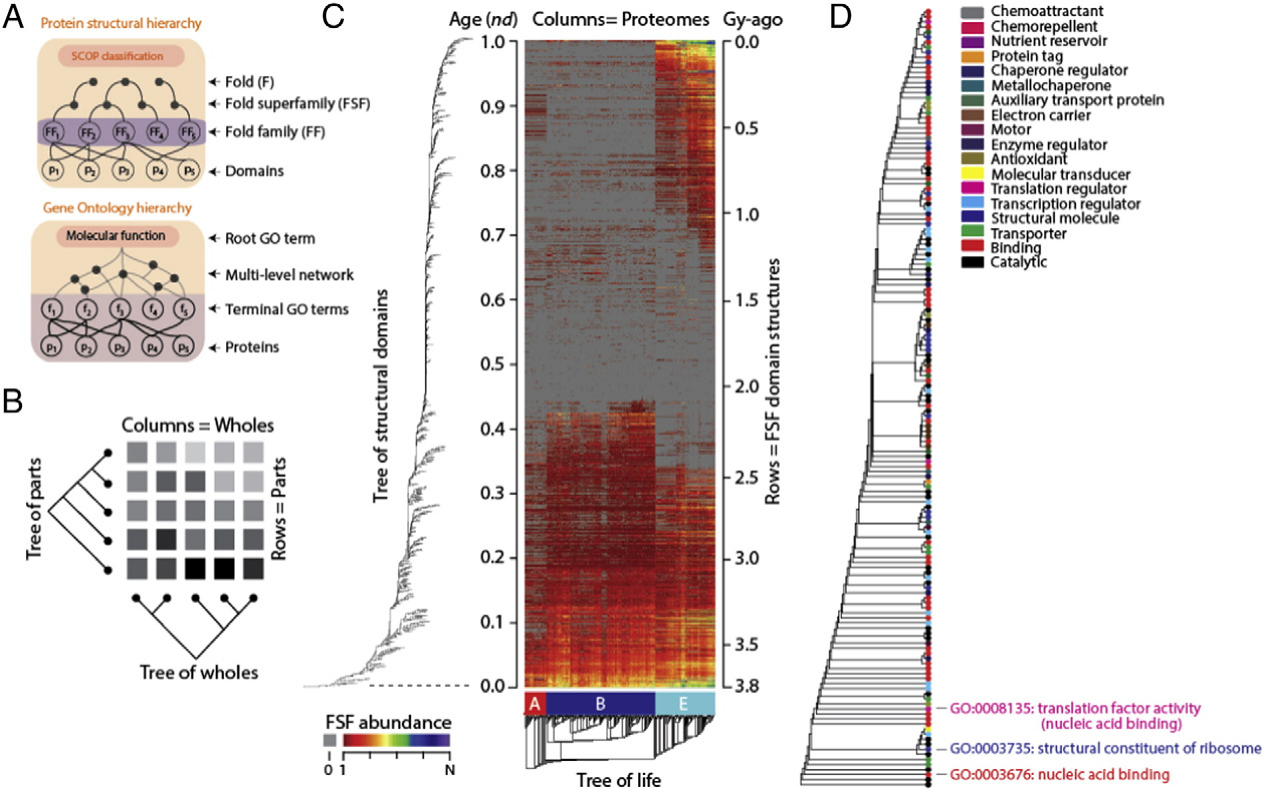
Exploring the evolution of proteins and molecular functions at global level. A. Taxonomies of protein domain structure and their associated functions hold a hierarchical structure. For example, higher SCOP or GO levels define structures and functions at lower granularity and deeper evolutionary abstraction level. B. The evolution of parts and wholes of biological systems can be explored by identifying useful features in parts and wholes (*phylogenetic characters*; Table 1) and by building matrices of qualitative or quantitative descriptions of those features (illustrated with boxes with different shades). These matrices permit the construction of both trees of parts and wholes by simple matrix transposition operations (Table 1), which describe their evolution, utilizing the tools of phylogenetic reconstruction. Note the comb-like appearance of trees of parts (which permits building timelines) and the more balanced appearance of trees of wholes. C. An evolutionary heat map of abundance of protein structural domains at SCOP FSF levels of abstraction in the proteomes of 420 free-living organisms defines a data matrix that is used to construct rooted trees and timelines (timetrees) of structural domains and rooted trees of proteomes (i.e. trees of life) describing the origin and evolution of Archaea (A), Bacteria (B) and Eukarya (E) [46]. Gray cells in the matrix imply an abundance of 0 (absence of the structure). Red-to-blue hues represent increasing abundance levels, from 1 to 15,112 counts of a same FSF structure. Ages of FSF in the timeline are time-calibrated with a global molecular clock of fold structures that spans 3.8 billions of years (Gy) of planetary history. D. Tree of level 2 GO terms of molecular functions (MF), with leaves colored according to level 1 GO classification. Selected ancient leaves are named and show the late appearance of nucleic acid recognition and genetics (nucleic acid binding) and ribosomal structure and specificity (structural ribosomal constituents and translation factors) following the early onset of metabolism. Data from [68].

The world of protein sequences (sequence space) remains uncharted. However, the number of higher-level structural modules can be considered finite. A number of protein classification schemes are available that are based on the structural similarity and common evolutionary origin of structural domains. Two of them, the Structural Classification of Proteins (SCOP) [26] and the CATH database [32] group proteins into a similar hierarchy of structures. For example, SCOP is a molecular taxonomical resource of high quality that groups domains in fold families (FFs), fold superfamilies (FSFs), folds and protein classes [26]. Domains with pairwise amino acid sequence identities of more than 30% are unified into FFs, and those FFs that share similar structures and functions are further unified into FSFs, which most likely have common evolutionary origins (Fig. 1A). FSFs sharing common core topologies (similar arrangements of secondary structures in 3D space) are further unified into folds, and those that share similar overall designs or properties (e.g. mostly helical or mostly strand) are further unified into protein classes. SCOP currently describes known structures in Protein Data Bank (PDB) entries with about 1200 folds, 2000 FSFs and 4000 FFs. These numbers are small relative to other classification schemes, which atomize molecular structure to levels closer to the sequence level (e.g. the Pfam database [33]).

Despite decades of effort, the systematic classification of protein structure remains limited by the lack of a general metric for global pairwise (or multiple) comparisons that can unify the widely different structural fold topologies [34]. This ultimately stems from our ignorance of the evolutionary forces responsible for molecular structure and structural innovation. However, Emile Zuckerkandl, one of the founders of the molecular evolutionary field, was well aware that the world of proteins could be unified despite the existence of an ensemble of patchy structural groups [8]. He also understood, as Eck and Dayhoff [1] did, that the process of molecular innovation was gradual and complied with the principle of continuity that supports biological evolution. “The chances for evolving proteins and protein classes (read fold structures) de novo are further increased at very early evolutionary stages on account of probable primitive characters such as smaller size, reduced complexity, and reduced specificity of interactions. It is obvious, for instance that a small and essentially uni-functional structure … will have higher chance of arising de novo than a large, multifunctional structure” [8]. Recent efforts are now advancing understanding of biophysical constraints that are acting on evolution of protein sequence, structure and function (reviewed in [35]). Importantly, during this past decade a shift of focus from molecules to proteomes provided global evolutionary views of the protein world that were aligned with the ‘shared and derived’ tenet of phylogenetic analysis [5], [36]. We will review some of these studies, which exploit the power of cladistics to uncover patterns of macromolecular accretion in proteomes, making unique links to geology and biophysics.

The turning point that enabled the evolutionary unification of the world of protein structural domain was the recognition that a structural census of their occurrence or abundance in genomes carried deep phylogenetic signal and could be used to track the origin and evolution of proteins and proteomes. Gerstein [37] used distance-based methods to build trees describing the evolution of proteomes from the occurrence of fold structures in genomic complements available at that time. Caetano-Anollés and Caetano-Anollés [38] later on used strict cladistic methods to extend the approach to genomic abundance of structural domains. Since then, ‘trees of life’ of these kinds have been reconstructed from genomic occurrence and abundance of domain structures (e.g., [39], [40], [41]) and from surveys of domain organization in proteomes [42], [43], confirming that domain structure carries significant evolutionary information. Beginning with Caetano-Anollés and Caetano-Anollés [38], studies polarized character transformation sequences (see *character polarization*, Table 1) to root the phylogenomic trees and avoid the need of outgroups (basal taxa used a priori as rooting hypotheses). The approach recognized that the abundance of domains in genomes is a costly trait to develop; each and every new domain variant of a same fold structure takes millions of years to unfold by gene duplication, subfunctionalization and neofunctionalization. Once accumulated, these structures are almost impossible to lose in evolution as they spread in cellular networks (e.g. the widely popular TIM α/β barrel superfold of metabolism) and establish numerous interactions. Consequently, their counts represent linearly ordered (additive) character states of multistate character transformation sequences that are ‘forward’ polarized [38]. Ordered states imply a Euclidean distance relationship of costs (transformation costs of two non-neighboring states are always larger than one step). Forward polarization imparts under *Weston's generality criterion* (homology in nested patterns [44]; Table 1) the property of rooting phylogenetic trees and identifying through polarization's ‘evolutionary arrow’ the origin of proteins and proteomes. Technically, optimal unrooted trees can be rooted a posteriori by using the *Lundberg rooting* method (Table 1) or by considering a hypothetical ancestor as reference. Assumptions of forward polarization have been validated by a number of criteria, and recently highlight the importance of using realistic evolutionary models in phylogenetic analysis [45].

Caetano-Anollés and Caetano-Anollés [38] also recognized that useful phylogenetic characters, such as the abundance of fold structures, could be used to build not only ‘trees of wholes’ but also ‘trees of parts’ (Fig. 1B). A simple transposition of the character matrix (*matrix transposition*, Table 1) used for reconstruction of *trees of proteomes* (rooted trees of wholes approximating ‘trees of life’) allowed reconstruction of *trees of structural domains* (proteomic parts), rooted trees that describe at global level the evolution of proteins. Since then, the ‘transposition’ strategy has been extended to the study of domain structure at all levels of protein classification, using both SCOP and CATH definitions (reviewed in [46], [47]). Fig. 1C showcases the approach in a recent analysis of domain structure at the FSF level of the SCOP structural hierarchy [46]. The heat map visually illustrates abundance levels of the data matrix used to build trees of wholes and parts, with columns and rows ordered according to the trees. The ordered matrix already uncovers interesting patterns of FSF use and reuse in the three superkingdoms of life that suggest an ancient stem line of descent [46].

Trees of parts are comb-like (pectinate) in appearance. Their highly unbalanced distribution of *nodes* (Table 1) is a consequence of accumulation of an evolving heritable trait. Chronologies describing the appearance of structural domains can therefore be constructed directly from the unbalanced trees of domains by calculating a “node distance” (*nd*), the relative number of internal nodes from the root to a leaf of the tree (Fig. 1C). Since much of protein history has occurred in cells, even prior to episodes of cellular diversification, chronologies capture history of protein structures in proteomes from the origin of modern proteins (*nd* = 0) to the present (*nd* = 1). Remarkably, a global molecular clock of domain structures calibrated with biomarkers and geomarkers revealed a significant linear relationship between *nd* (the age of domains) and the geological record (in billions of years, Gy) [48]. This places the origin and evolution of modern biochemistry within a framework of planetary history.

Chronologies of structural domains have been used to study the origin and evolution of modern metabolism [49], [50], [51], the natural history of its biocatalytic mechanisms [52], the impact of oxygen [53], [54], metallomes and biological metal utilization [55], the origin of translation [56], [57], and the coevolutionary history of the ribosome [58] and the specificity of the genetic code [59]. Chronologies consistently identified enzymes of nucleotide metabolism harboring the P-loop containing triphosphate nucleoside (NTP) hydrolase fold as points of origin of the protein world (e.g. [38]). They revealed that the first domains capable of interacting with RNA were catalytic domain of aminoacyl-tRNA synthetase (aaRS) enzymes that aminoacylate tRNA. They appeared late, together with GTP-binding domains of initiation and elongation factors harboring the P-loop NTP hydrolase fold 3.7 Gy-ago, well after the acylating domains of non-ribosomal protein synthetases (NRPS) [56], [57], [58]. Ribosomal protein domains appeared later again supporting an origin of translation is non-processive mechanisms of protein biosynthesis. Remarkably, clustering of evolutionarily conserved ‘persistent’ genes in genomes delimited three concentric rings of gene neighbors [60]. The discontinuous and loosely connected outer ring was the most ancient. It included genes devoted to metabolism, which encircled the other two rings that were organized around translation. The second ring harbored aaRSs and the most recent central ring revolved around ribosomal proteins and information processing. Thus, both the structure of domains and physical clustering of genes in genomes provide congruent views of the evolutionary accretion of proteins in proteomes.

**Fig. 2:**
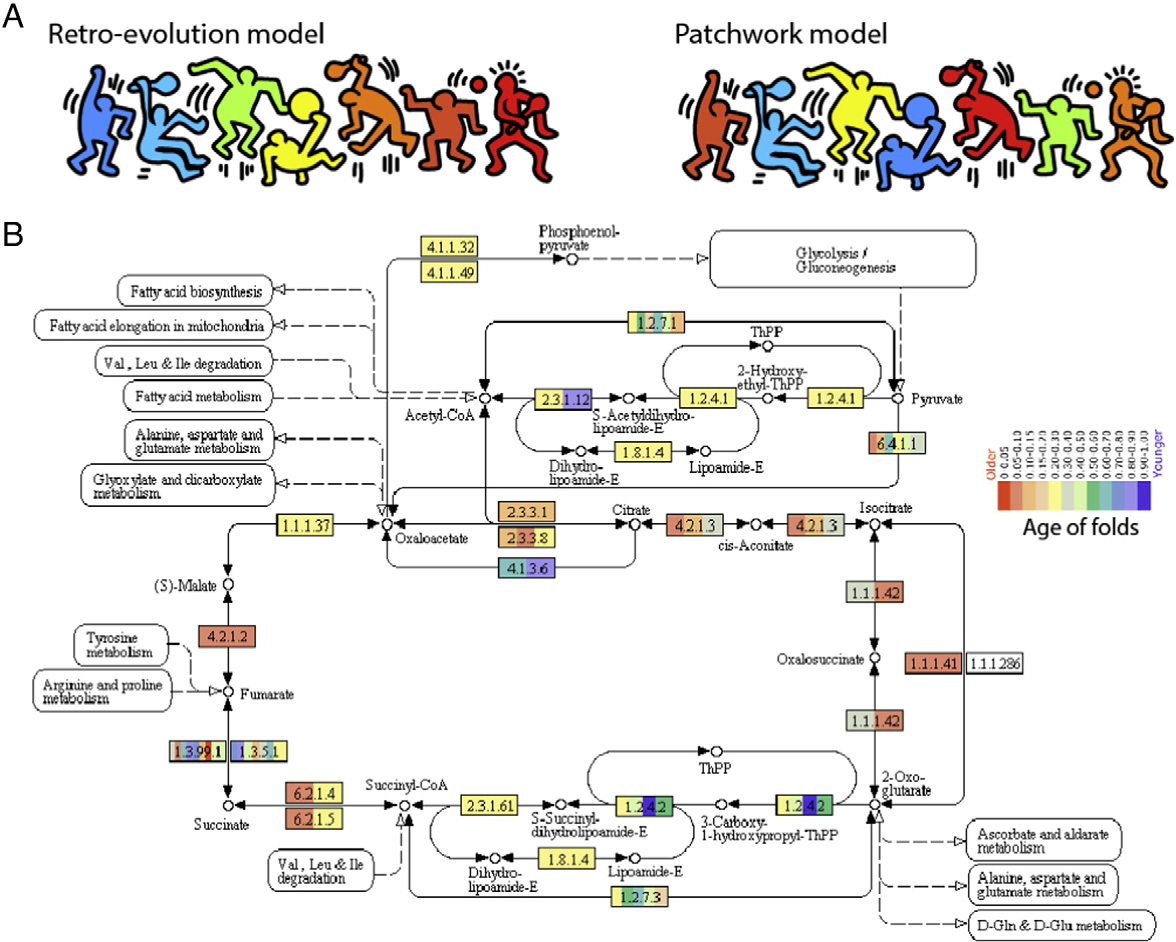
Metabolic network evolution. A. Accretion of enzymes in pathways can follow two main evolutionary models, which are illustrated with colored Keith Haring-inspired figures (enzymes) exchanging balls (metabolites) (artwork by D. Caetano-Anollés). The retro-evolution model stresses the gradual, ordered and systematic addition of enzymatic elements. The patchwork models allows for enzymes to be recruited freely and result in a mosaic of ancestries. B. The citric acid (TCA) cycle subnetwork (CAR 0020) of MANET 3.0 shows patchwork patterns. Domain structures associated with individual enzymatic activities (described in EC nomenclature) are painted according to their age, in a scale of node distance (*nd*) that ranges from 0 (the oldest enzymes) to 1 (the most recent) using a tree of protein structural domains defined at FF level of the SCOP hierarchy.

Chronologies also helped uncover patterns of enzymatic accretion in metabolic networks [49], [50], [51]. Several processes responsible for metabolic evolution have been proposed, including specialization of multifunctional enzymes, pathway duplication and divergence, pathway retroevolution, and enzymatic recruitment. Two hypotheses that explain the growth of metabolic pathways have been the most popular, the retro-evolution (retrograde) and the patchwork model (reviewed in [61])(Fig. 2A). Horowitz [62] proposed the influential retro-evolution scenario, in which biosynthetic (anabolic) pathways evolve backwards. The first enzyme to appear is the last in a pathway, and pathways gradually coalesce into an evolving network of metabolic reactions. The driving force is the model is gradual depletion of successive organic molecules, which poise the rise of new metabolic activities and substrate intermediates. In contrast, the patchwork model assumes the existence of promiscuous catalytic activities that are recruited pervasively and fortuitously to perform different functions [63], [64], [65]. This results in a patchwork (mosaic) of homologous enzymes spread throughout metabolic pathways. Considerable nomothetic and ideographic evidence supports the primacy of the patchwork model. For example, assignments of fold structures to enzymes of the metabolic network of *Escherichia coli* revealed a genuine mosaic structure (e.g. [66]). Similarly, the systematic tracing of domain age in metabolic networks revealed a patchwork of ancestries [50], [67]. Both approaches showed there was little repetition of structures or ages of enzymes in consecutive enzymatic steps. Fig. 2B shows a metabolic subnetwork of the MANET database with the age of enzymatic domains traced on enzymatic functions defined by Enzyme Commission (EC) classification. The example subnetwork and others reveal a patchwork of ages and structures spreading throughout metabolic networks, which clearly dominates the difficult-to-identify retro-evolving (and forward-evolving) patterns. The retrograde and patchwork models make explicit two competing evolutionary modes of molecular accretion, which are recurrent in molecules and molecular repertoires: (i) *a gradual mode*, in which ordered growth is facilitated by a simple and dominant mechanism, and (ii) *a patchwork mode*, in which unordered growth is dictated by multiple processes that sometimes interact with each other in frustrated manner. The gradual mode is often responsible for ‘core-periphery’ or concentric layering patterns, which can be strongly diluted by the patchwork mode.

## 4 Accretion of molecular functions and the origin and evolution of functionomes

Since molecules are the main effectors of molecular functions and biological processes, they hold important ontological information. From a philosophical point of view, this information is necessary to understand the basic principles that drive biology. From a computational point of view, there is also need to appropriately name and annotate molecular and cellular entities. This is necessarily associated with the concept of information and the definition of parts and wholes, modules and biological hierarchies. The exercise also requires development of tools for classification and taxonomies. The Gene Ontology (GO) database represents a community effort to unify biological annotations of molecular functions (MF), biological processes (BP) and cellular components (CC) [68]. The scheme is hierarchical and similar to protein structure classification (Fig. 1A). However, the tree-like structures are organized into a multi-level hierarchy of ontological terms that often involve reticulations. Three independent directed acyclic graphs (DAGs) describe MF, BP and CC annotations, in which child GO terms associate with multiple parents to account for functional relationships (links between terms) and associations (links between terminal GO terms and genes). We note that the GO hierarchy approximates an evolutionary hierarchy [69]. Higher-level GO terms (e.g. level 1 or level 2) are more encompassing and ancient while lower-level terms (e.g. level 4 or terminal terms) are more modern. This notion is inspired by the proposal that functions that are promiscuous can serve as evolutionary ‘starting points’ for more specialized functions [63], [64], [65], [70]. The concept of functional promiscuity being ancient is for example supported by the primacy of the patchwork model of metabolic evolution we discussed above. The link between DAG structure and evolution therefore justifies the use of GO terms as phylogenetic characters and the application of the ideographic framework to the study of repertoires of molecular functions (functionomes).

Indeed, using the part and whole paradigm and the matrix transposition strategy described above, we were able to reconstruct *trees of functionomes* and *trees of functions* [69], [71], [72]. These trees carry considerable phylogenetic signal and are reconstructed directly from a genomic census of GO terms defined at different levels of the DAGs and at different stringency levels. They describe the evolution of functionomes and their component parts. For example, Fig. 1D shows a tree of level 2 MF terms, with leaves colored according to level 1 GO annotations (from [69]). The tree makes once again evident the accretion process responsible for modern functionome makeup. The most basal MFs were metabolic functions (hydrolase and transferase activities) [69]. These ‘catalytic’ functions preceded ‘binding’ functions (nucleic acid binding), and these preceded ‘structural’ (structural constituents of the ribosome) and ‘regulatory’ functions (translation factor activity nucleic acid binding). The tree of MFs of Fig. 1D is therefore in line with evolutionary patterns derived from the structural phylogenomic census of structural domains previously described. These patterns support the late interaction of proteins with nucleic acids and the late appearance of the ribosome in evolution observed for example in the phylogenomic analysis of FSF [56]. Phylogenies at lower GO levels showed the primordial appearance of ATPase, GTPase and helicase activities [69]. This was confirmed by a subsequent analysis of terminal GO terms that made ATP binding the most ancient term at this lowest level of the GO hierarchy [72]. Finally, trees of GO BP terms showed that cellular biopolymer metabolic processes preceded biopolymer biosynthesis and essential processes related to the formation of macromolecules [69], challenging again the existence of a ‘replicator-first’ paradigm and supporting instead a ‘metabolic first’ origin of life. It is now clear that the phylogenomic study of abundance of structural domains and GO functions result in congruent views of molecular accretion.

## 5 Accretion of helical components in RNA

As we discussed above, the ‘abundance’ (popularity) of cellular component parts, when suitably defined, carries deep historical information. Abundance also holds the ‘arrow of time’. The popularity of molecular structures and functions increases in time at global levels, as parts diversify and comply with both the principle of continuity and gradual growth. Domain abundance in proteomes for example increases via de novo creation, gene duplication, cooption and rearrangements, ensuring valuable innovations are not easily lost by mutation. In fact, the history of character changes has been retraced in phylogenomic trees built from occurrence and abundance of structural domains [73]. The character state reconstruction exercise shows that the number of domain gains overshadows the number of losses throughout the timeline of protein evolution. This confirms the fundamental trend of accretion, despite pervasive losses that occur across lineages and throughout evolution.

RNA macromolecules also carry deep phylogenetic signal [74], [75], [76] and the arrow of time in their structures [77], [78], [79]. RNA base pairs associate and disassociate at rates < 0.5 s^− 1^ [80]. Furthermore, RNA folding rate is dependent on chain length [81]. By assuming that the distribution of free energy barriers separating the folded and unfolded states is Gaussian (supported by the central limit theorem and polymer biophysics), the rate of folding of RNA was found to be negatively correlated with polymer length and its speed limited to not more than ~ 1 ms. These findings fit experimental rates of folding. Consequently, the frustrated kinetics and energetics of the folding and collapse process allows for only some conformations to reach stable states [82]. RNA structure evolves by reducing the number of possible conformations that can form so that their average life is sufficiently long to hold durable molecular functions [83]. The thermodynamic stability of evolved molecules also increases in a process known as ‘structural canalization’ [84], [85]. This global trend to increase molecular persistence and stability can be exploited in phylogenetic reconstruction to produce rooted phylogenies of parts and wholes, taking advantage of considerable background knowledge from cladistics, morphometrics and statistical mechanics [77], [78], [79].

Geometrical and statistical features of substructures such as helical stems or loops commonly found in RNA are surveyed in thousands of RNA molecules, coded into linearly ordered and polarized multi-state characters, and the resulting character state matrices used to build trees of molecules (wholes) and trees of substructures (parts) (methodology reviewed in [86]). The phylogenetic model automatically roots the trees by assuming conformational stability increases in evolution as structures become canalized (a posteriori using the Lundberg or the hypothetical ancestor rooting approach). For example, statistical characters such as the Shannon entropy of the base-pairing probability matrix or features of thermodynamic stability (e.g. minimum free energy of conformation, barrier energetics) define a morphospace that imparts polarization. Evolutionary models like these are supported by considerable evidence (e.g. molecular mechanics, simulations, phylogenetic analysis, thermodynamics; [86]) and comply with Weston's generality criterion through positional and compositional correspondence. The method has been applied to the study of a number of molecules, including rRNA [58], [77], [78], 5S rRNA [87], tRNA [79], [88], RNase P RNA [89], and SINE RNA [90], to study molecular evolution of closely or distantly related organisms spanning years (e.g. continental introduction of a plant pathogenic fungus [76], ascomycete population differentiation [91], or coral evolution [92]) to billions of years of evolution (rise of superkingdoms; e.g. [77], [78], [79]).

**Fig. 3:**
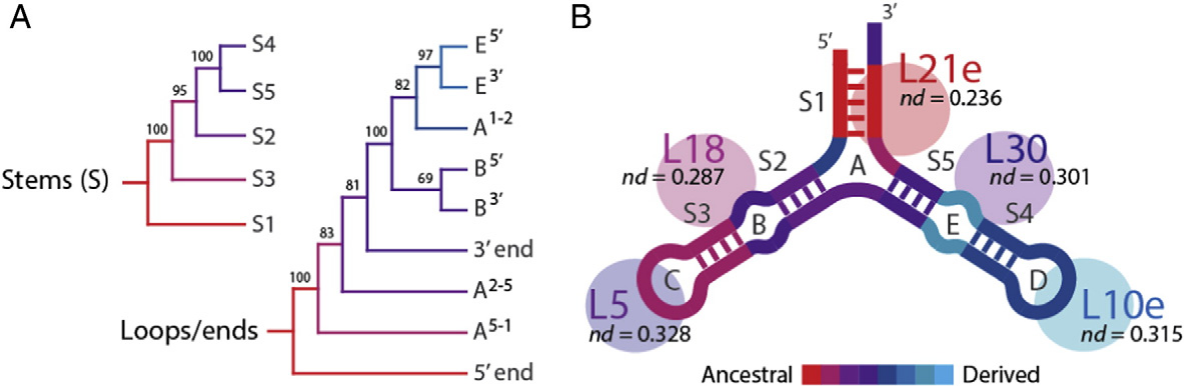
The natural history of the structure of 5S rRNA. A. Rooted phylogenetic trees of stem and loop/end substructures illustrate the evolutionary statements reconstructed from structural information. These phylogenies represent models of molecular growth. Similar trees (not shown) describe the evolution of hairpin structures, bulges and G:U pairs. Bootstrap values > 50% are shown for individual nodes. B. Consensus evolutionary model of the 5S rRNA molecule showing the relative ages of individual RNA substructures traced on its secondary structure. The age of ribosomal proteins interacting with different sections of the molecule is given in node distance (*nd*). Increasing *nd* values represent the progression of evolutionary time. The relative ancestry color scale describes the number of nodes from the hypothetical ancestor at the base of the tree of substructures. Data from Sun and Caetano-Anollés [80].

Trees of RNA substructures are particularly valuable because they explicitly describe the evolutionary accretion of molecular parts. Building trees of paired and unpaired substructures of 5S rRNA, for example, enable the construction of chronologies of the accretion process (Fig. 3A). Since deeper branching substructures of the comb-like trees are more ancient, the ancestrality of substructures can be represented with color scales that can be traced onto 3D structural representations of the RNA molecules (Fig. 3B). Phylogenies reveal that helix S1 (domain α), especially its 5′ end, represents the origin of 5S rRNA. S1 is the most basal helix of the molecule. Terminal helix S3 of domain β is the second oldest and structures of domain γ are more derived. Growth of the 5S rRNA molecule matches the establishment of interactions with ribosomal proteins, suggesting coordinated evolution (coevolution) of ribosomal proteins and RNA [87]. Accretion of 5S rRNA shows patchwork patterns of accretion, with outward (towards the periphery) and inward (towards the core or base of the molecule) growth episodes occurring throughout its history. Interestingly, tree reconstructions exclude a possible single ancestral duplication responsible for the y-shaped molecule [93] supporting instead a more gradual model of growth [94]. We note that accretion of RNA substructures must occur within the context of higher-order 3D structure, interactions established with other molecules, and the crucial selective backdrop of molecular function.

## 6 Exploring ribosomal accretion

Explaining how complex RNA molecules form by gaining atomic structure is paramount for synthetic biology but challenging. Large molecules such as the rRNA molecules of the ribosome can be considered collectives of A-form helices joined to each other by multi-branch loops known as RNA junctions. Junctions are pivot points connecting two or more helical stem regions [95]. They act as crossroads defining the structural organization of helical RNA and the overall tree-like abstraction of complex molecules [96]. Junctions play important structural and functional roles, especially in complex ribonucleoprotein complexes. They constrain RNA dynamics by enabling structural flexibility (e.g. [97]) and by interacting with proteins [98]. In fact, molecular dynamics simulations confirm chromophore-based energy transfer studies, revealing that three-way junctions act as flexible ribosomal elements [99]. At atomic level, most loop nucleotides form an ordered array of non-Watson–Crick base pairs that is also structurally constrained [100]. These constraints define different topologies, which allow the grouping of junctions into families [101], [102], [103]. For example, two helices stack coaxially in most three-way junctions. Therefore, these junctions can be classified according to the configurations of helical components: perpendicular (family A), diagonal (family B) and parallel (family C) [101], [102], [103]. Similarly, coaxial stacking interactions and spatial alignments of helices group four-way junctions into nine families [102]. Higher-order junctions also contain typical coaxial helical stacks and parallel or perpendicular alignments of helices [103].

The ribosome is an essential molecular machine that is universal in cellular organisms. It distinguishes cells from viruses. The main structural ribosomal components are the small and large subunits. The small subunit (SSU) typically contains one rRNA molecule (16S/18S rRNA) holding ~ 50 universal helices that fold independently into three major domains [104]. The structure harbors the ‘decoding’ center that enables the reading of mRNA information by the anticodon loop of tRNA and the central mechanism of the turnstile, the ribosomal ratchet. The large subunit (LSU) typically contains 2–3 rRNA molecules (23S/28S and 5S/5.8S rRNA) with ~ 100 universal helices that fold into six domains (5S rRNA is the seventh) [105]. The structure holds the peptidyl transferase center (PTC) that is responsible for protein biosynthesis and structures specialized in ribosomal mechanics and energetics, such as the L1 and L7/12 stalks, the GTPase center, and the α-sarcin–ricin loop (SRL), needed to move the tRNA molecules through the central groove of the complex. Both subunits associate by establishing inter-subunit bridges [106]. The small and large subunits also hold about 30–40 and 30–45 ribosomal proteins, respectively, and interact with a host of factors that mediate ribosomal energetics and specificity [107]. While rRNA constitutes the bulk of the ribosome, proteins stabilize the complex and play multiple additional roles.

**Fig. 4:**
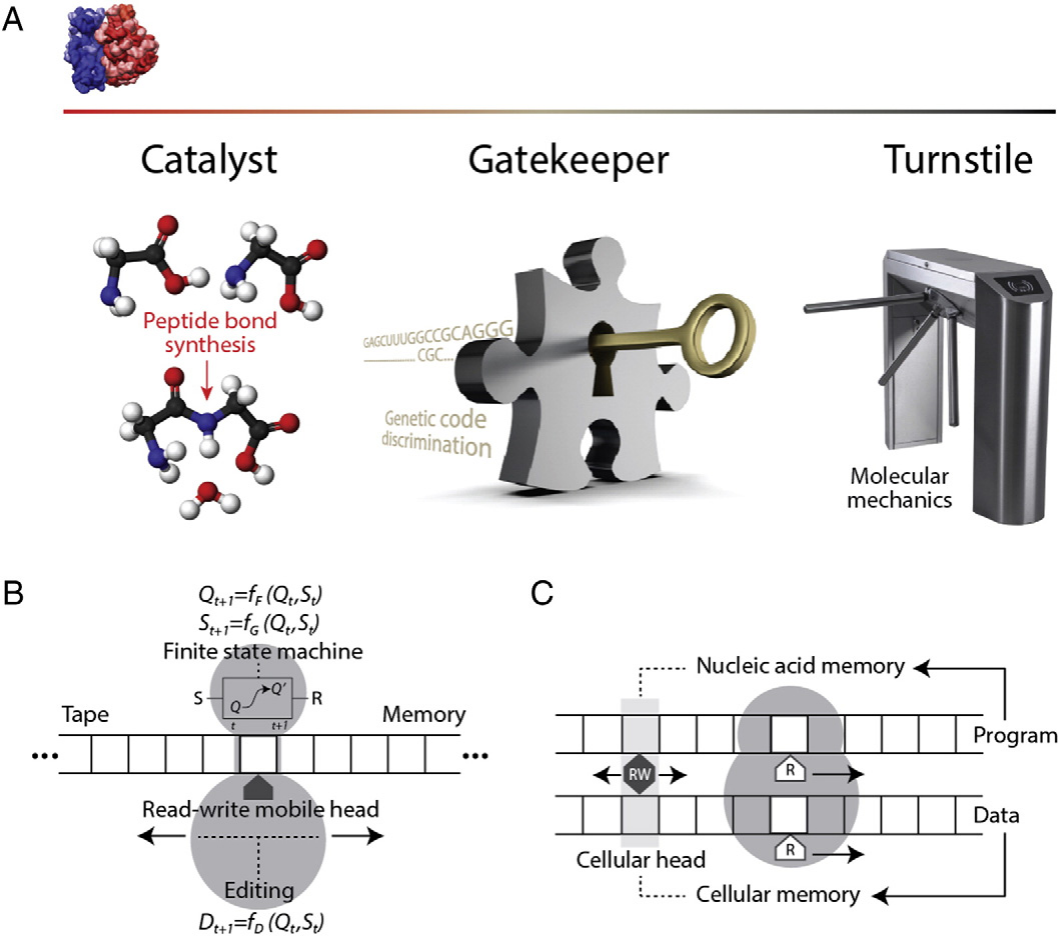
Ribosomal roles for biological computation. A. The three main roles of the ribosome involve the synthesis of peptide bonds, the ability to discriminate aminoacylated tRNA, and the movement of RNA through mechanical gates and switches that resemble a turnsile. B. A Turing computational machine has both finite state machine and editing behaviors. When a finite state machine (a black box) receives a stimulus (input S) it changes its internal state *Q* to *Q′* generally through a non-linear response R. Two functions of both input symbols S and states *Q* describe the machine. Given a tape with placeholders for symbols of an alphabet, the finite state machine can operate recursively through a mobile head. If this head has editing capabilities, it can read-and-write (RW) symbols and can move left or write in the tape according to an additional function, turning the finite state machine into a Turing machine. C. A universal Turing machine can have two tapes, one storing the ‘program’ for reading machines (current state and symbol and description of machines with sets of functions *S, Q* and *D*) and the other for storing the data of machines. In the universal ribosomal Turing machine the program tape defines the nucleic acid memory and the data tape stores the memory of polypeptide machines. Both move left during biosynthesis through read-only (R) turnstile heads. The RW ‘cellular head’ operates at different timescales and in multiple cells through selective optimization and driven by cellular persistence.

The origin and evolution of the ribosome have been always mysterious and have prompted a multitude of hypotheses. Many origin scenarios continue to be inspired by the ancient RNA world theory (e.g. [108], [109], [110]). These scenarios generally place the origin of the complex in its catalytic center. However, the ribosome holds several functional roles besides being a *catalyst* for the synthesis of peptide bonds (Fig. 4A). The ribosome is also a *gatekeeper*, policing genetic code discrimination during decoding, and fundamentally, a *turnstile* capable of molecular mechanics and information and energy transfer. Which of these three general ribosomal roles in cells is uniquely universal, central and likely ancient? A cursory analysis provides preliminary clues:

(i) The ability to catalyze peptide bonds is not unique to the ribosome (reviewed in [57]). NRPS modules provide an assembly-line system capable of producing peptides with cyclic and branched structures from hundreds of atypical amino acid building blocks. Catalytic domains of class I and II aaRSs can also form peptide bonds as cyclodipeptide synthetase enzymes of diketopiperazine biosynthetic pathways and as truncated forms in antibiotic biosynthesis and aminoacylation of carrier proteins. Peptide ligase enzymes harboring a variety of folds (e.g. ATP grasp, SAICAR synthase-like, Acyl-CoA N-acyltransferases) are also involved in peptide biosynthesis. Most of these domains appear earlier than ribosomal proteins in timelines of structural domains, but none before the catalytic domains of aaRSs. Since ribosomal proteins are central for the functioning of the ribosome, phylogenetics suggests that catalytic synthesis of peptides unfolded earlier than in the PTC of the ribosome [57].

(ii) Translation specificity is the modern ‘memory’ of the genetic code that manifests when information is transferred from nucleic acids to proteins. However, ribosomal discrimination against non-cognate tRNA substrates is at least 100–1000 times less stringent than that of aaRS protein enzymes and elongation factor (EF) binding [111]. Consequently, the ribosome is not the main gatekeeper of genetic information. Such roles are entrusted to protein structural domains of aaRSs and factors, which are older than ribosomal proteins [57].

(iii) In contrast, the turnstile mechanical properties of the ribosome that have been recently uncovered are remarkably complex and unique [112]. Its structural components are also likely very ancient. In the process of *ribosomal translocation* (Table 1), the million-dalton ribosomal complex moves ~ 10 Å along mRNA in each codon step prompted by successful interactions between the universal ternary complex of aminoacylated tRNA and EF.GTP and the decoding center. This movement overcomes a 80–100 kJ/mol activation energy barrier between a ‘pre’ and ‘post’ translocation state of the complex with tRNA bound at the A and P sites or P and E sites, respectively. In the pre-translocation state, SSU and LSU subunits rotate against each other by 7°. This ‘ratcheting’ movement is accompanied by a fully reversible and GTP-independent movement of tRNAs occurring *only* in LSU, from A and P sites into P and E sites. Since no analogous movement occurs in SSU, tRNAs are forced into A/P and P/E hybrid SSU/LSU sites. Importantly, the rotation is associated with a strong 30° inward movement of the L1 stalk of the LSU from an ‘open’ to a ‘closed’ position, causing a 50 Å displacement of the stalk tip that acts as a gate of the E-site. The binding of EF^.^GTP to the pre-translocation ribosome induces a strong conformational change in SSU, ‘swiveling’ its head 30° inward towards the E site and inducing the ‘pre’ to ‘post’ transition together with the triggering of SRL-mediated EF-dependent GTPase activity. The rotation of the head involves flexing of two crucial hinges in the neck between the head and body of SSU [113], both of which involve very old rRNA structures (h28 and h34 [58]; see below). The mechanical movement of the turnstile is tightly coupled with the decoding process of the A site, which matches the first two base-pairs of the codon-anticodon duplex via a number of sequence-independent hydrogen bonds established with conserved positions of SSU rRNA (including h44, the ribosomal ratchet) and ribosomal protein S12. Structures supporting these interactions contribute to the stability of the pre-translocation step and the energetics of the activation barrier [112] and are the oldest components of the ribosome [57], [58]. The turnstile operation is also tightly coupled with the movement of the anticodon arm of tRNA from the P site to the E site through the so-called ‘A760 gate’ in SSU and to h44 nucleotide intercalations in mRNA that prevent its back-sliding during head rotation. As we will later describe, the ribosomal turnstile and its collective of at least three gates and one switch (its logical operation) is a *finite state machine* (Table 1) for use in universal biological computation (Fig. 4B and C).

This cursory analysis suggests that the highly conserved structures of the complex turnstile mechanism are very old and should be considered candidates for ribosomal molecular origins. Indeed, the accretion process of the complex has been made explicit in detail using ideographic studies that suggest the ribosome originated in its processive decoding and mechanical functions [58], [78]. However, a series of recent nomothetic studies contend the ribosome originated in the PTC and its protein biosynthetic function. We now describe the findings of these studies and limitations, especially those highlighted in very recent exchange of correspondence [114], [115], [116], and the implications for origins of biochemistry.

### 6.1 Ideographic analyses of ribosomal origins and evolution

Phylogenies and evolutionary timelines of ribosomal history were recently derived from an analysis of thousands of RNA molecules and millions of protein structural domains using the tools of phylogenomic analysis described above [58]. The study was well supported by preliminary exploration [77], [78], [87], [117]. In initial studies, Caetano-Anollés [77], [78] reconstructed trees of life from the structure of SSU and LSU rRNA and traced the complete repertoire of structural characters lineage-by-lineage in the trees. Because the trees were rooted, the age of the substructures that were traced along the branches revealed several remarkable patterns [78]. Patterns of character change showed there was an overall tendency towards molecular simplification as rRNA structures grew. Tracings also uncovered evolutionary patterns of inter-subunit bridge contacts and tRNA binding sites that were consistent with the coupling of tRNA translocation and intersubunit movement, including the concerted evolution of tRNA binding sites in the two subunits. Remarkably, crucial functional structures such as those participating in protein synthesis and translocation were older compared to other structures, revealing already the ancestrality of ribosomal dynamics. A later and more encompassing study of the evolution of the entire ribosomal complex using trees of substructures confirmed the suspicion that the central SSU rRNA ratchet (helix h44) was the most ancient structure of the ensemble [117].

**Fig. 5:**
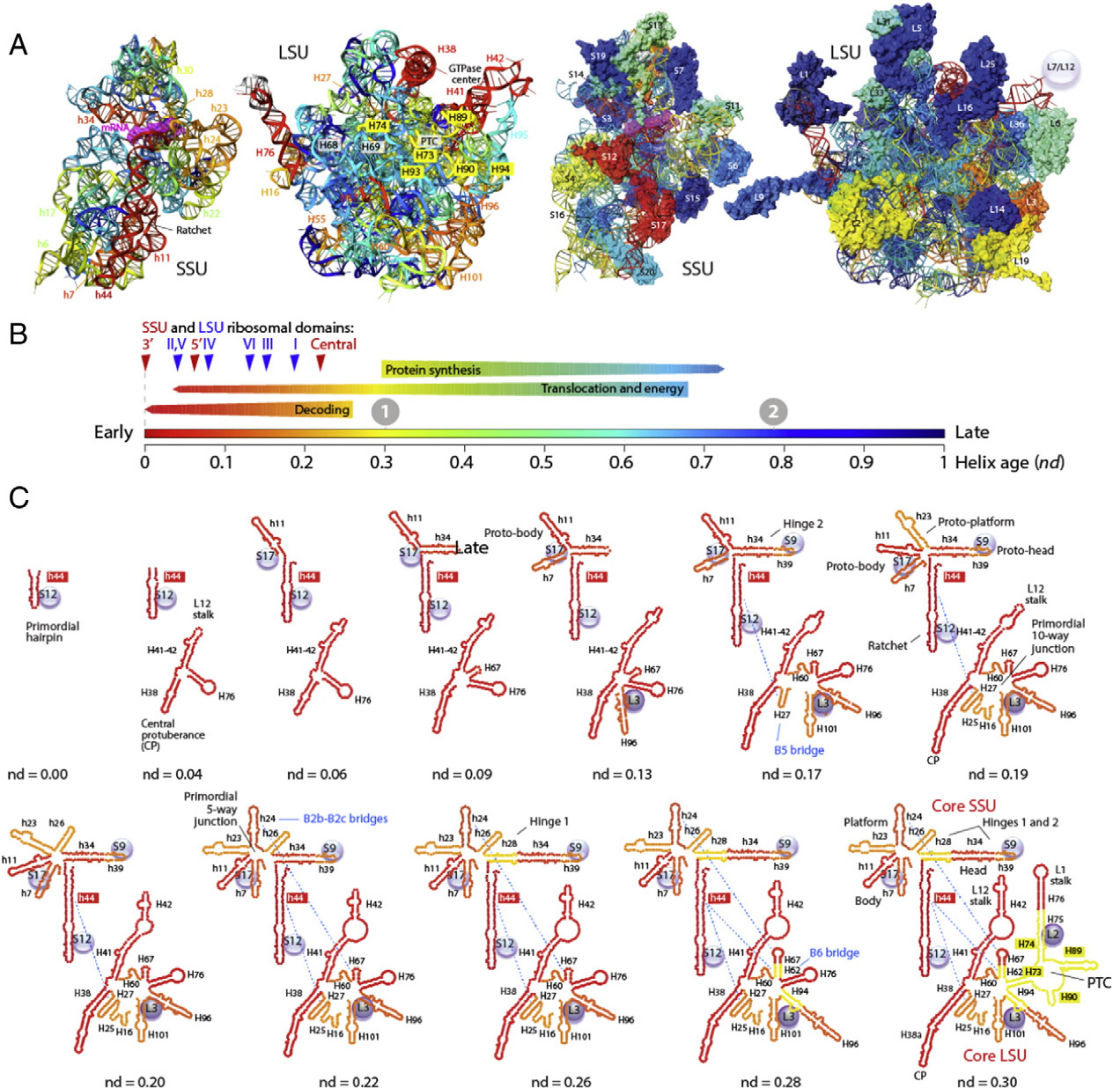
The natural history of the ribosome derived from phylogenomic analysis of protein structural domains and RNA structure. A. Evolutionary heat maps of *Thermus thermophilus* SSU and LSU crystal structures (PDB entries 2WDK and 2WDL) with helices and interacting ribosomal proteins colored according to their age (*nd*) [57]. B. Timeline describing the early appearance of ribosomal domains and global functions (tRNA translocation and GTPase associated energetics, mRNA decoding and helicase activity, and protein synthesis). The first and second evolutionary transitions are indicated with encircled numbers. C. Step-by-step model of ribosomal accretion leading to the first evolutionary transition of ribosomal history, the formation of the peptidyl-transferase center (PTC) and primordial ribosomal cores (data from [57]). Secondary structures are colored according to age and growth of helical segments modeled with growth rates of 100 bp/*nd* (~ 26 bp/Gy) and an average start length of 15.9 ± 11 (SD) bp to assume recruitment. Bridge interactions are indicated with blue dashed lines and proteins with labeled buttons.

Encouraged by coevolutionary patterns discovered between ribosomal proteins and 5S rRNA [87], Harish and Caetano-Anollés [58] embarked on a comprehensive study of the history of the entire ribosomal complex. The relative ages of structures of ribosomal proteins and RNA drawn directly from the phylogenetic trees were indexed with structural, functional and molecular contact information and mapped (by color) onto three-dimensional models of the ribosome (e.g. rRNA; Fig. 5A). A molecular clock of folds [48] was used to place relative ages of proteins (and indirectly RNA) onto the geological record. Several remarkable evolutionary patterns were uncovered arising directly from the reconstructed timeline (Fig. 5B). First, timelines showed the coevolution of ribosomal proteins and RNA components in both ribosomal subunits. The oldest protein (S12, S17, S9, L3) appeared concomitantly with the oldest rRNA substructures responsible for decoding and ribosomal dynamics (SSU helices h44, h11, h34 and LSU helices H38, H41, H76) 3.3–3.4 Gy-ago (Fig. 5). Proteins-RNA coevolution continued throughout the timeline. Second, the appearance of RNA substructures at first occurred in orderly fashion until the LSU and SSU structures formed central and open 10-way an 5-way junctions, respectively (Fig. 5C), but later on became increasingly patchy (Fig. 5C). Third, a major transition in ribosomal evolution that occurred 2.8–3.1 Gy-ago brought ribosomal subunits together through inter-subunit bridge contacts that stabilized the loosely evolving ribosomal components. During this transition, a fully-fledged PTC with exit pore capable of protein biosynthesis appeared by duplication of local helices, supporting an appealing model of PTC origin [108], [110]. Protein L2 also made its appearance at this time, presumably to help L3 stabilize the newly formed PTC core. Fourth, a second evolutionary transition occurred almost concurrently with the “great oxygenation event” of the planet (ca. 2.4 Gy-ago) and involved the accretion of the L7/12 protein complex that stimulates the GTPase activity of EF-G and enhances ribosomal efficiency. Finally, the global accretion process continued until the present by adding additional structural layers of junctions, including the extension of a growing peptide tunnel. Following the first evolutionary transition, numerous tertiary interactions (e.g. A-minor motifs [118]) were established, especially in the LSU rRNA core [36] that respected the order of appearance of the interaction partners. It is possible that these additional interactions increased the stability of the complex and its processivity, culminating in the second ribosomal transition.

The study also revealed that tRNA was at the center of ribosomal evolution. The major transition involved not only deployment of the PTC and bridges but also interactions with a clover leaf-like tRNA molecule in newly developed A, P and E sites (see additional analyses in [119]). tRNA–rRNA interactions occurring before the transition involved ancient SSU helices and the modern half of tRNA (including the AC loop). tRNA–rRNA interactions occurring after the transition involved newer LSU helices and the older half of tRNA. Appearance of interactions with the TΨC arm immediately after the emergence of the PTC makes the arm the only tRNA region capable of interacting with the two ribosomal subunits, confirming the evolutionary centrality of tRNA structure for ribosomal evolution.

It is important to reiterate that timelines are ideographic models derived from phylogenies that are supported by both genomic and molecular data and the phylogenetic model of character state transformation (the tripartite tree-data-model paradigm). These timelines are not the products of imagination, but the result of discovery operations of *Hennigian illumination* (Table 1). Timelines must be permanently revised by increasing the amount of data (genomes and molecules) that are useful, enhancing the structural classification of protein structural domains and RNA elements used as characters, and providing additional support to the phylogenetic model (see discussions in [59]). Timelines are also supported by nomothetic background knowledge, including knowledge about the structural makeup and mechanics of ribosomal components. When tracing the age of structural components of modern ribosomes, structural inaccuracies of structural models should be considered inconsequential to the validity of phylogenetic inferences. These structures represent placeholders of evolutionary knowledge. For example, assume that a new ribosomal structure is discovered that contains an additional helix. The age of this helix cannot be traced until the genome and corresponding ribosomal molecules are included in the dataset. However, its existence will not affect the conclusions and validity of previous studies. Similarly, discovery of new molecular processes and functions and growing phylogenetic knowledge interact synergistically, helping each other.

### 6.2 Nomothetic analyses of ribosomal evolution

Sober and Steel [120] raised the important issue of the epistemic relation that connects the present to the past and how researchers use ‘traces of the past’ to reconstruct biological history. They caution about the dangers of considering these traces as unerring information, especially in light of the Markov Chain Convergence Theorem (MCCT) and the Data Processing Inequality (DPI) that describe how information between the present and the past is affected by time. In their analysis, the ‘optimistic’ conclusion that the present would provide strong evidence about the past can only be reached if there would be a natural process that connects the past to the present (i.e. a *Laplacian demon*; Table 1) and there would be a one-to-one mapping of the states of the biological system in the past and in the present. Mathematically, they confirm information never completely disappears in processes that involve simple population models and zero mutation, even after infinite time. However, many other models cannot guarantee the one-to-one mapping nor they can avoid the eroding effects of time. Nomothetic thinking assumes that ‘living fossils’ (tree rings) directly inform about natural processes and guarantee accurate recovery of history. However, living fossils, much as their non-living counterparts, may not represent ancient ancestors but ancient relatives. Their historical traces can degrade and be the subject of the same MCCT and DPI limitations that complicates ideographic analyses. In the absence of phylogenetic reconstruction, nomothetic models cannot test the effects of time and their historical validity.

Despite caveats of these kinds, a series of nomothetic studies constructed maps of helical-stack interactions in A-minor motifs [121], concentric shells structures [122], and branch-to-trunk insertions [123] in rRNA of the large ribosomal subunit. Without support from phylogenetic analysis and inspired by the ancient ‘RNA world’ hypothesis, studies assumed directly or indirectly that the ribosome originated in the PTC and that extant molecular interactions, putative accretion shells, and rRNA insertions were sufficient to portray evolutionary change. The origin of the ribosome was therefore established a priori through algorithmic implementations, and its validity left untested, trusting that the models of molecular growth that were used truly represented universal Laplacian demons.

#### 6.2.1 A model based on A-minor interactions and periphery–core ribosomal dismantling

Bokov and Steinberg [121] used tertiary interactions in the LSU rRNA molecule to build an algorithmic model of evolution of the large ribosomal subunit of *E. coli*. Their nomothetic model makes use of properties of the components of the A-minor motif, an interaction of a helix with an adenosine stack that together with other motifs [124] is widely present in LSU rRNA [118], [125]. The conformational integrity of the adenosine stack depends on the presence of the interacting helical segment of the motif. In turn, the stability of the helix does not depend on adenosine packing, since there are many ribosomal helices without A-minor motifs. This forms the basis of an interesting ancestral–descendent relationship proposition for molecular origins. However, careful study makes it evident that A-minor motifs cannot dissect relationships between all structures of the LSU rRNA molecule. Additional information was required, which took the form of the core–periphery paradigm. The nomothetic study was therefore based on three fundamental assumptions (in order of importance): (i) the order of appearance of structures must respect a base-to-apex directionality imposed by ‘local insertions’ responsible of outward (apical) molecular growth (dependency D1); (ii) helices appear before the adenosine stacks in evolution (dependency D2); and (iii) rRNA is considered a circular molecule. Operationally, the extant bacterial ribosome was subjected to 12 rounds of systematic dismantling and elimination of structures that could be tagged by dependencies of the D1 (56 statements of growth by apical insertion) and D2 (59 A-minor motifs) type. Dismantling started from apical structures considered the most recent ribosomal additions and proceeded backwards in time. Helices that were apical to substructures were dismantled first to maintain a core-to-periphery structural layering and a succession of putative insertions that extended the molecule through outward growth. When A-minor interactions were not present, the order of elimination was made from apex to base (basipetally) by claiming that the elimination of the basal segment would split the circular molecule into two segments, i.e. the existence of a D1 dependency. The resulting model of ribosomal growth showed that the central, L1, and L7–L12 protuberances supporting translocation and the GTPase reaction were evolutionarily derived. This was expected since the algorithm forces peripheral layers to become late molecular additions. The model also showed that LSU rRNA originated in the PTC, and that the ancient core was gradually stabilized by addition of increasingly peripheral structures. However, the periphery-to-core back-in-time dismantling algorithm [121] gradually collapses the six domains of the LSU rRNA molecule into its central supporting junctions, making these core portions ancient. The central core includes the 10-way junction that unifies all structural domains and the 5-way junction supporting the PTC of domain V. The algorithm cannot dissect unequivocally which dismantled structure is periphery and which is core, and the exercise becomes highly subjective and flawed [116]. For example, sorting out the last three layers of elimination, which depend on only two A-minor motifs, provides many evolutionary scenarios that are equally likely (Fig. S1). More importantly, there are numerous possible scenarios of elimination that would produce different LSU rRNA origins that are worthy of becoming central structural cores (some described in Fig. S1). Furthermore, the algorithm allows for structures of the central, L1, and L7–L12 protuberances supporting ribosomal mechanics to be points of origin, if peripheral structures are allowed.

Despite the fundamental flaws of the terminal algorithmic steps, there is significant evolutionary signal in A-minor motifs. The phylogenomic study of Harish and Caetano-Anollés [58] mapped ages of structural partners in the A-minor interactions of SSU and LSU rRNA and found that the majority of helices (~ 80%) evolved before the A-stack, supporting assumption (ii) of the algorithmic model (see Fig. 4 of [36] for a map of A-minor interactions in LSU rRNA). Perhaps the minority of non-complying A-minor motifs result from the early appearance of both unpaired and helical tracts, which then associate at a later time to stabilize the junction structures that were unfolding in the ensemble. This possible outcome was never discussed by Bokov and Steinberg [121] and further complicates the validity of their model.

#### 6.2.2 A model based on branch-to-trunk directionality of apical insertions

In contrast with the previous approach, Hsiao et al [122] utilized a strategy that was completely aligned with the core–periphery paradigm. Inspired by a previous proposition [126], they explicitly assumed that the origin of the LSU rRNA and the ribosome was the PTC. They then converted LSU rRNA structures of *Thermus thermophilus* and *Haloarcula marismortui* into ‘onions’ by sectioning 10 Å-thick concentric shells centered in the PTC, modeled as a sphere. They found that sequence and conformational conservation between the structures was maximal near the PTC and diverged gradually towards the periphery. This trend was seen as confirmation of an origin of the molecule in its main catalytic center.

**Fig. 6:**
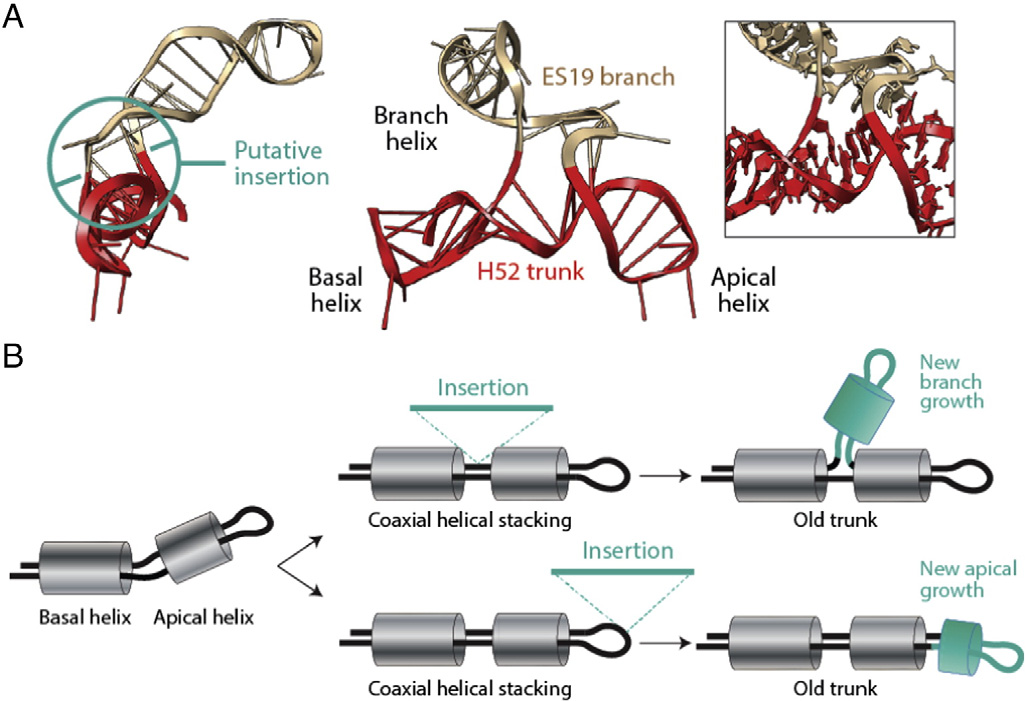
rRNA expansion mediated by branch-to-trunk insertions. A. Helix H52 of the *Saccharomyces cerevisiae* ribosomal model (colored red) overlaps almost perfectly with homologous helices in bacterial and archaeal rRNAs. However, the yeast structures have an extra branch (ES19, colored tan) that is probably the result of an insertion. Lateral and coaxial views of the H52–E19 structure were visualized using ‘ribbon’ backbones with ‘ladder’ sugar-base configurations to better visualize the coaxial layout. The inset shows molecular details of the insertion using ‘fill–fill’ sugar-base configurations. An ‘insertion fingerprint’ was defined as a change inducing minimal distortion of trunk segments at branch sites, bases paired and stacked in trunks on either side of the branch point, linear or quasi-linear trunk axes, acute deviation of trunk–branch helical axes, and close apposition of trunk sugar and phosphate moieties. B. Cartoon describing the model of insertion of new branch structures into old trunk structures used by Petrov et al. [107] to trace back-in-time the evolution of the ribosomal core. Helical segments are portrayed with cylinders and unpaired regions with coils. A terminal RNA structure with a basal and apical helix gains with time coaxial helical stacking properties that further stabilize the overall helical structure. Sequence insertions in RNA coding genes can elongate these structures at terminal and internal positions of rRNA (aqua coil segments linked to insertion sites) if they do not affect the function or stability of the molecule. For example, an insertion into the unpaired region of a 2-way junction (or directly into the helical stem, see panel A) produces a branching structure (3-way junction) and forces acropetal (base-to-apex) growth of the molecule. Alternatively, a terminal insertion extends the unpaired region of a hairpin and forms an additional helical segment (aqua-colored cylinder) of apical growth.

The same group and collaborators recently took the ‘onion’ approach and coupled it to the Bokov and Steinberg model of ribosomal growth [123]. Comparison of atomic structural models of bacterial and archaeal ribosomes (e.g. [127], [128]) with recent eukaryotic structures [129], [130] revealed that new eukaryote-specific helical segments inserted into old common (universal) core LSU rRNA regions without significantly perturbing local helical conformations [117]. Structural alignments of bacterial and eukaryotic core ‘trunks’, i.e. trunks belonging to a universal ribosomal core, defined for example at secondary structure level [131], [132], showed minimum distortions of helical conformations, highlighting evolutionary conservation at 3D structure level. Fig. 6A shows an example of a putative insertion of a yeast-specific branch (tan colored) onto a core defined by helix H52. The constricted connection between the coaxially stacked basal and apical helices (the trunk) and the branch outgrowth was assumed to indicate a ‘trunk-to-branch’ time directionality imposed by the insertion of the new branch onto an older trunk [123]. These so-called ‘insertion fingerprints’ provided the conceptual foundation for a new algorithmic model of molecular growth (Fig. 6B). Formation of helical segments, sometimes subtended by two-way junctions, is followed by coaxial stacking interactions stabilizing the entire helical structure. Insertions of segments in either unpaired regions of the junction or in helical segments that do not destabilize the coaxially stacked helical arrangement are allowed to expand the growing rRNA molecule. These outgrowths are defined as ‘ancestral expansion segments’ (AES) of the growing structures. Analyses of several organism-specific rRNA junctions of recent origin supported the model of molecular growth [123].

**Fig. 7:**
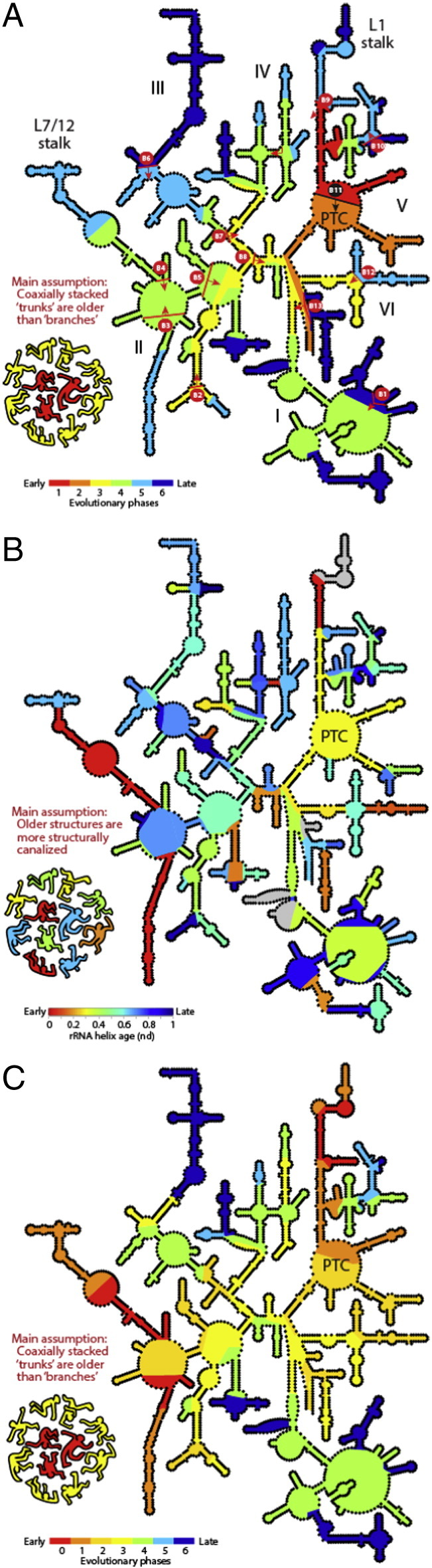
Origin and evolution of LSU rRNA. A. Insertion-based evolutionary model [107]. B. Phylogenomic-based evolutionary model [57]. C. Revised insertion-based model accounting for branch-to-trunk insertion misannotations of the original model (corrected in Fig. S2). The evolutionary age (*nd*) or evolutionary phase is traced by color on individual helical segments defined by the structural model of *Escherichia coli* LSU rRNA (PDB entry 3R8S resolution 3.0 Å; [110]) and represented as secondary structures. Putative insertions with misannotated branch-to-trunk directionalities are indicated with red lines and labeled with alphanumerical red identifiers. The putative insertion of the PTC is indicated in black (B11).

Assuming that the ancient ribosomal core evolved via similar mechanisms throughout its history, a series of putative insertions of new ‘branch’ helices onto preexisting coaxially stacked ‘trunk’ helices were further proposed, which originated in the PTC [123]. Fig. 7 shows an updated secondary structure model of LSU rRNA from *E. coli*, which is considered analogous to that of the ancestral ribosomal core. Helical structures are colored according to phases or ages of ribosomal history inferred from nomothetic trunk-to-branch directionalities [123] (Fig. 7A), and, for comparison purposes, from phylogenomic analyses [58] (Fig. 7B), respectively. In contrast to the phylogenomic model, which identifies that the most ancient structures are those supporting translocation mechanisms, the branch-to-trunk insertion model appears to identify the P-site-containing half of the PTC (AES 1–2) as the origin of the large subunit. This initial structural nucleus then accretes in orderly fashion a number of structural layers to form the ribosomal ancestral core. These layers correspond to six evolutionary phases: *Phase 1:* Formation of a branch duplex and P-loop with archaic protein biosynthetic abilities; *Phase 2:* Maturation of the primordial PTC by addition of the A-site and formation of the exit pore; *Phase 3:* Extension of the exit pore to form an early peptide tunnel; *Phase 4:* Acquisition of the SSU interface and reinforcement of the PTC and exit tunnel; *Phase 5:* Acquisition of the energy-driven translocation machinery (L1 and L7–12 stalks, central protuberance) and further extension of the tunnel; and *Phase 6:* Late extension of the tunnel and accretion of surface structures.

Remarkably, both the insertion-based [123](Fig. 7A) and phylogenomic-based [58](Fig. 7B) models share important features: (i) Ancestral and burst-like appearance of the PTC region (red/orange shades in the insertion model and yellow in the phylogenetic model), (ii) gradual addition of layers to a growing exit tunnel (yellow shades in the insertion model and green shades in the phylogenetic model); and (iii) overall 3D layering from a central core. However, the two models differ substantially in the evolutionary placement of the translocation machinery (late in the insertion model and early in the phylogenomic model) but also in the way helical segments are accreted. The insertion-based model reveals molecular growth spreading outwards, concentrically and continuously from the origin of the molecule. Instead, the phylogenomic-based model reveals a patchwork-like distribution of the ages of helices throughout the structure, reflecting an inward-and-outward model of molecular growth that is more complicated (Fig. 7B). The phylogenomic model also reveals that originating ancient structures spread throughout all the domains of rRNA, suggesting they were pushed towards the molecular periphery away from the base of the molecule (helix H1). Examination of tracings of distances spanning different parts of the LSU rRNA molecule and the PTC core (Fig. 2 in [117]) show that in general the apices of growing branches are located towards the molecular periphery. This tells little about how the structures grow, i.e. if they are pushed towards the periphery by outward or inward growth.

In search for extra clues buried in insertion fingerprints, we reanalyzed branch-to-trunk directionalities present in rRNA structure [114], [116], focusing on the list of 64 putative insertions and associated AES of LSU rRNA that were described and using the structural criteria employed by the authors (Table S3 of [107]). The 3D atomic structure of each junction of the LSU structure was examined, exploring coaxial helical patterns supporting trunks and studying the junction architectures with published network interaction diagrams defined by the Leontis–Westhof symbology [100]. As anticipated by the authors, many insertions, including all insertions located in 2-way junctions that involve ‘helix elongations’, “do not leave distinctive structural fingerprints” [123], and were therefore uninformative. However, our analysis revealed that 12 out of the 64 insertions had incorrect (conflicting and unjustified) branch-to-trunk assignments. These incorrectly annotated insertions are indicated with red lines in the secondary structure model and are labeled alphanumerically (Fig. 7A). Fig. S2 shows their insertion fingerprints and associated helices. Red-colored and tan-colored segments represent trunks and branches, respectively. The misannotations have significant consequences for the insertion-based model, when the model is revised to accommodate the branch-to-trunk reversals (Fig. 7C). Three misannotated insertions are particularly crucial. The insertion spanning AES1 (harboring H75, which provides basal support to the P-site of the PTC) and AES39 (harboring coaxial H76 and H79 stems with translocation functions) forms a typical ‘family A’ 3-way junction [101]. We found that the helices of AES39, and not the long AES1 branch (H75, H74 and H89), were part of the older trunk (Figs. 7 and S2, insertion B9). Similarly, the AES22–23 and AES14–16 insertions belonging to the 7-way junction that supports the central protuberance (CP) and L7/12 stalk were incorrectly annotated. Branch-to-trunk directionality reversals made the coaxial stacks of AES23 (H41 and H45) and AES16 (H37 and H38) older than the rest of the multi-loop structure (Fig. 7, insertions B3 and B4). These three crucial misannotations involving trunks associated with both ribosomal translocation and energetics have the consequence of adding an additional older phase to the insertion-based model, *Phase 0* (colored deep dark red in Fig. 7C). The new additional phase holds the very early development of all translocation structures of LSU rRNA, crucially reconciling both insertion-based and phylogenomic models of ribosomal evolution.

Despite congruence, we note important limitations. The six-phase (Fig. 7A) and revised seven-phase model (Fig. 7C) cannot be recovered unambiguously from ancestral insertion data. Dissecting pathways of accretion becomes increasingly complicated as trunk–branch relationships unfold from the origin(s) of the molecule towards the increasingly branched molecule [114]. In the absence of an objective algorithmic function capable of dissecting this difficult conceptual and combinatorial problem, any attempt to fit insertion data into non-phylogenetic models derived from accretion shells [122], A-minor interaction networks [121], or other extant structural information, will fail to objectively test detailed models of ribosomal origin and accretion. This can be illustrated with revisions induced by reversals of malafide branch-to-trunk directionalities in domain III, spanning insertions B6 and B7 (Fig. 7C). The region is layered at roughly constant distance from the PTC (Fig. 2 in [123]) and does not hold A-minor interactions or other features of interest [121] that could dissect a plausible evolutionary progression. This makes assignments to evolutionary phases subjective.

#### 6.2.3 Patterns of coaxial helical stacking in rRNA highlight the complexity of the evolutionary model of molecular growth

**Fig. 8:**
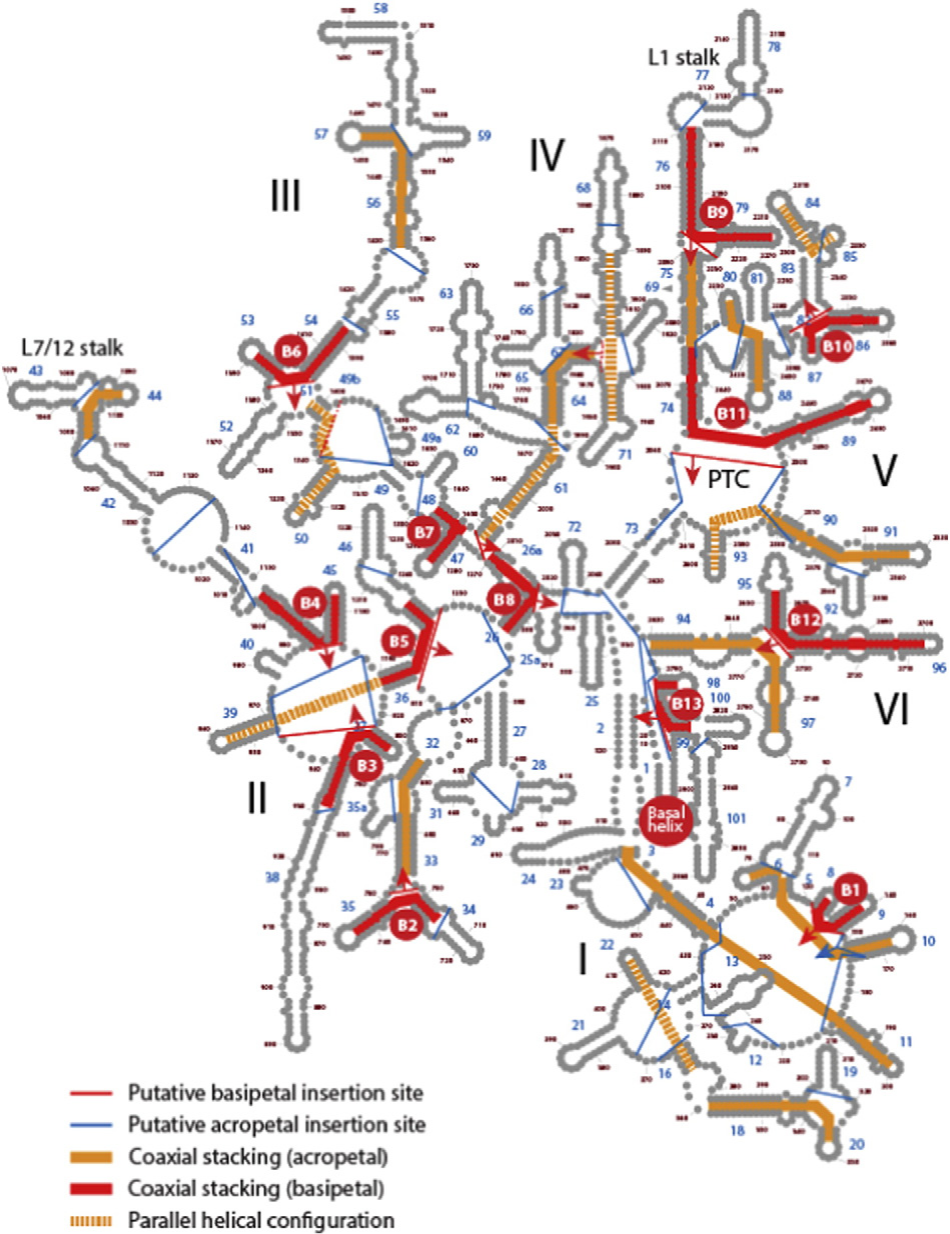
Patterns of coaxial helical stacking and putative insertions in rRNA support an origin of the large ribosomal subunit in structures that enable translocation mechanics. A secondary structure of the large subunit of the *Escherichia coli* ribosome, revised according to its high-resolution rRNA structure (Fig. 7), was annotated with unambiguous coaxial helical stacking regions [93], [94] and putative sites of insertions [117] involving 3-way and higher order junctions. Coaxial helical stacking regions exhibiting basipetal and acropetal branch-to-trunk insertion directionalities are colored red and orange, respectively. Basipetal directionalities are indicated with arrows. Stacked helices in B3, B4 and B9 subtend fundamental structures supporting translocation mechanics and stacked helices B11 subtend half of the PTC. Patterns of coaxial helical stacking falsify an origin of the ribosome in the PTC (see [115]).

Once branch-to-trunk misannotations are corrected, the nomothetic insertion-based model supports the origin of LSU rRNA in its mechanic functions (Fig. 7C), matching the phylogenomic-based proposal [58]. Once again, the corrected model falsifies an origin of the ribosome in the PTC. However, the insertion-based method rests on the validity of the algorithm of sequential apical insertions and outward growth. This model *must be universal*, in sharp contrast with phylogenetic models, which manifest locally as trees/networks and molecular data are mutually optimized during phylogenetic reconstruction. We make explicit the limitations of the insertion model by tracing coaxial helical stacking in the secondary structure model of LSU rRNA (Fig. 8). This allows direct visualization of branch-to-trunk directionalities and how they distribute in the overall LSU rRNA structure. Out of 25 informative insertions (Table 2), about half of insertions are ‘basipetal’ (B1-to-B13, labeled in red), i.e. the branch (or branches) protruding from the trunk point inward towards the base of the molecule (i.e. helix H1). Each of these insertions suggests a separate instance of inward molecular growth that departs from the outward insertion model. Basipetal insertions spread evenly in the structure, providing coaxial helical stacking support to the most central and important junctions (e.g. B8, B9, B11), but also to peripherically located structures of LSU rRNA (e.g. B2, B10). Their presence in each and every domain of the LSU rRNA structure shows that inward growth is pervasive.

**Fig. 9:**
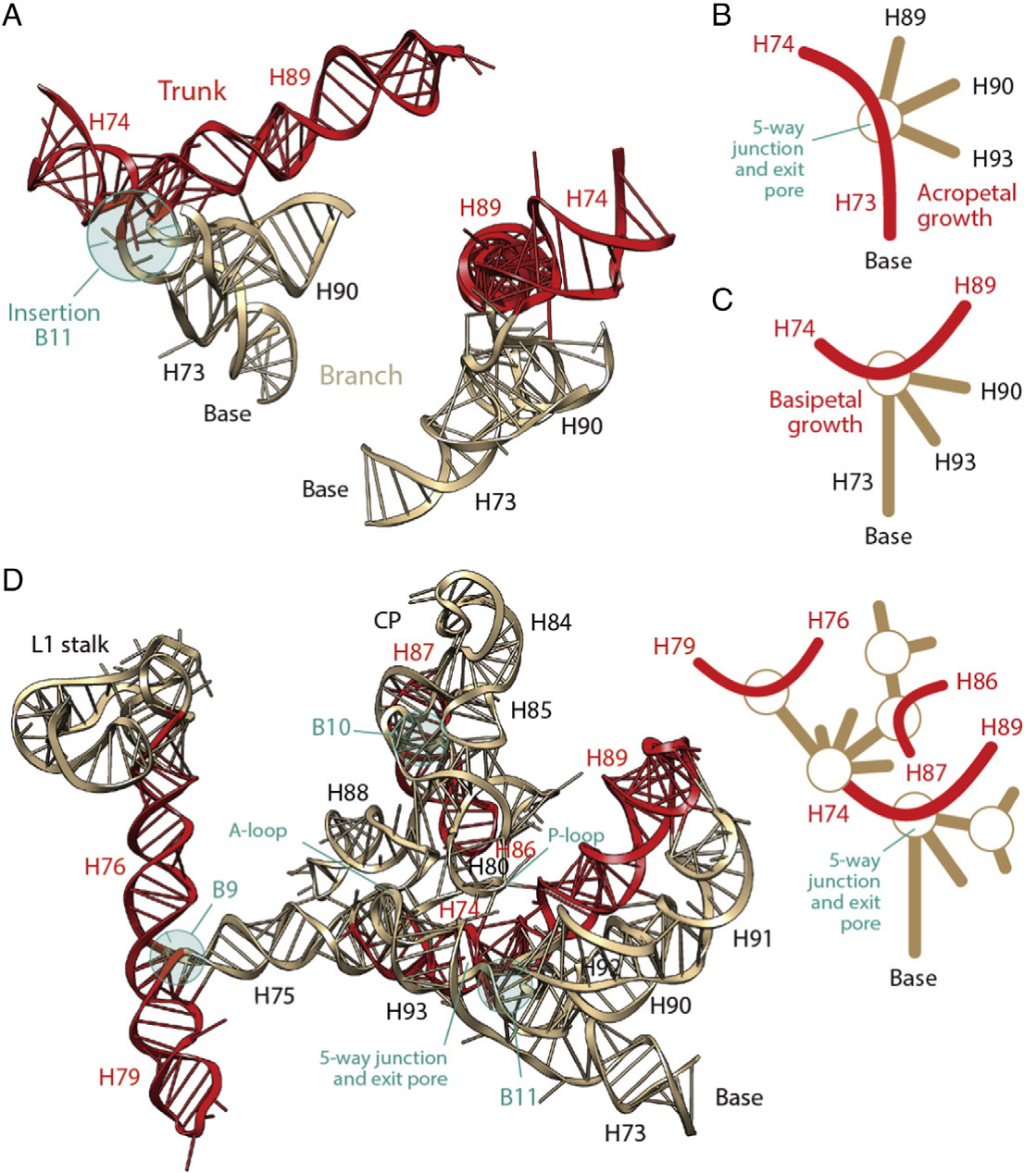
Analysis of domain V of LSU rRNA and the 5-way junction supporting the PTC. The P-site of the PTC is supported by helices H74 and H89 (A). These helices form a coaxially stacked trunk that is colored red in side and radial views of the substructures and hold a putative insertion (B11) responsible for the formation of H73 and H90. The acropetal model however expects that coaxial stacking establish between helices H73 and H74. Instead, coaxial stacking of H74 and H89 imposes a branch-to-trunk directionality that is basipetal and cannot leave an insertion fingerprint record (see description in text). This problem is made explicit in domain V, which holds two additional insertions with basipetal branch-to-trunk directionalities (B9 and B10) (D). The acropetal model cannot explain these coaxial helical patterns, which are made explicit by tracing them in cartoons of secondary structure models.

We illustrate the problem of the insertion-based model by studying the structures supporting the P-site of the PTC, the L1 stalk and its translocation functions, and the CP, all of which are located in domain V (Fig. 9). Helices H74 and H89, which are coaxially stacked, subtend the P-site. They form a trunk (part of AES1) that is topologically apical to the insertion (B11) proposed by Petrov et al. [123] that connects them to the base of domain V (H73) (Fig. 9A). The trunk is part of the 5-way junction that defines the PTC and the exit pore. If this AES1 trunk was primordial, H74 and H89 had to form an initial 2-way junction that was ‘open’ and harbored the 5′ and 3′ terminals. Under the insertion model of outward growth, apical insertions produce branch growths that can only increase the order of the PTC-containing junction. They cannot close the junction by adding helix H73. This requires separate 5′ and 3′ terminal insertions complementary to each other for pairing and formation of a closing helix, which cannot leave a record of the proposed B11 insertion fingerprint (Fig. 9A). Thus, the insertion-based model predicts a scenario (Fig. 9B) that is incompatible with the observed coaxial helical patterns of the 5-way junction harboring the PTC (Fig. 9C). A similar case can be made for insertion B9 connecting the L1 stalk to the rest of domain V, and for insertion B10, which defines a 4-way junction of the CP region (Fig. 9D). On an aside, we note that the Bokov and Steinberg model added the unrealistic assumption of a circular rRNA molecule to circumvent the problem that we now make evident in domain V.

**Fig. 10:**
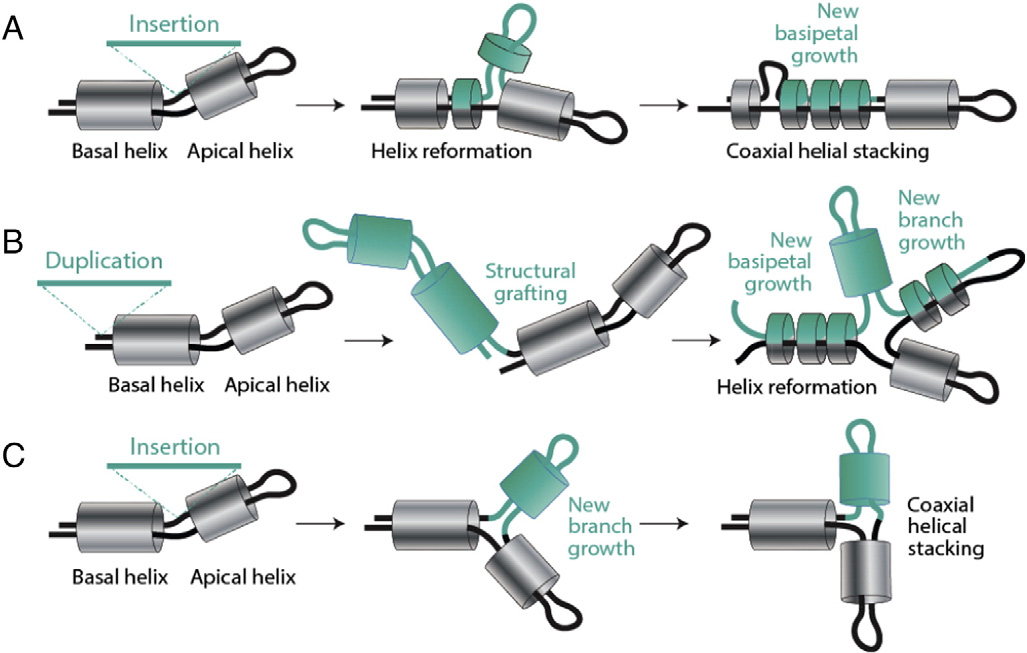
Models of basipetal (inward) ribosomal growth. The cartoons illustrate examples of structural changes induced by mutations, insertions and duplications that cause basipetal molecular growth (A and B) or apical growth with basipetal branch-to-trunk directionalities (C). Structural changes include helix reformation, helix stabilization by coaxial helical stacking, and topological rearrangements of the growing molecule. In contrast to models of apical insertion (Fig. 6), insertions in these models favor ‘basipetal’ growth, inward towards the base of the molecule. They can occur anywhere in the molecule (including deep basal paired and unpaired regions). Insertions can cause considerable rearrangements of existing structures by for example reforming base pairing (A). Reformations are less likely if the initial helices were initially stabilized by tertiary interactions. However, once a non-deleterious insertion is accepted by the organismal population, mutations slowly ‘canalize’ (sensu [82]) the reformed structures by constraining the conformational ensemble of the molecule and establishing stabilizing intramolecular interactions (e.g. coaxial helical stacking, psudoknotted structures and A-minor and ribose-zipper interactions; [106]). Basal tandem duplications produce ‘structural graftings’ (B). Base pair complementarities poise extensive reformations, which can preserve the stability of the original non-duplicated structure. While both acropetal and basipetal insertions can branch the molecule, tandem duplications have the ability to produce higher-order junctions. Branch outgrowths provide opportunities for increased flexibility, intra-molecular interactions, and stability. Finally, branch growth can occur in regions that have not established coaxial helical stacking (C). The development of stacking interactions with basal or apical helices then stabilizes the branch outgrowths. Interactions with apical helices however produce basipetal branch-to-trunk directionalities and signatures that deliberately hide the original acropetal insertion.

Failures to explain patterns of origin and evolution of each domain of LSU rRNA prompt a revision of the insertion model of ribosomal growth. Any instance of inward growth requires ‘helix reformation’, the restructuring of existent helical structures (Fig. 10A). Models of these kinds are not new. They have been proposed over two decades ago for the origin of the tRNA molecule by dimerization of primordial hairpin structures [133]. A simple tandem gene duplication event provides for example ample base complementary to form complex cloverleaf structures from hairpins (Fig. 10B). Such a model could explain the formation of the junction supporting the PTC (Fig. 9). Modeling has shown that helix reformation occurs by continuous and discontinuous transitions in sequence space [134]. Rare point mutations can result in helix formation and helical shifts that are discontinuous. These shifts sometimes require extensive reformations by mutation. In contrast, loss of helical tracts or extension-or-shortening of helical tracts are continuous transformations. Thus, major structural changes can occur through single mutations steps, while minor structural alternations may require extensive mutational exploration (reviewed in [85]). It is therefore likely that many genetic insertions will not leave insertion fingerprints and that many junctions will not be the result of apical growth but of helix reformation. Finally, new branch growths by insertion will at first produce malleable structures (expressing multiple suboptimal conformations), that are then ‘structurally canalized’ (sensu [84]) to produce for example coaxially stacked helices (Fig. 10C). This new insertion mode could explain coaxial helical stacking with inward branch-to-trunk directionalities. However, it weakens the algorithmic insertion model of ribosomal growth since it renders insertion fingerprints meaningless in absence of retrodiction.

Using the phylogenomic model, we traced the evolutionary age of helices subtending junctions with basipetal and apical coaxial helical stacking to test the validity of insertion models (Table 2). Remarkably, only two (B4, B7) out of 13 putative basipetal insertions had branches older than trunks and one (B11) had branches and trunks of equal age. This supports the appearance of branches before coaxial helical stacking (see model of Fig. 10C), falsifying the existence of insertion fingerprints in these junctions. Only four out of 12 putative apical insertions had trunks older than branches and two had branches and trunks of equal age. Thus, few apical insertions can be validated with the phylogenomic model. The data suggests a frustrated interplay of apical-basipetal dynamics that compromises, in the absence of phylogenetic information, the construction of an unequivocal insertion-based model of macromolecular accretion with nomothetic methods.

## 7 Evolutionary implications of molecular accretion patterns

What are the molecular evolution and origin-of-life implications of the reconstructed history of molecular accretion in proteomes and ribosomes? Phylogenomic data embedded in hundreds-to-thousands of proteomes provide strong support to an alternative model of origin of biochemistry, translation and early life that is different and has more explanatory power than the widely accepted ‘RNA world’ paradigm [36], [56], [57], [58], [59]. In this model, historical information in the structural domains of proteomes is ‘remembered’ by the biological system, even in the absence of modern genetics, and can be mined with standard phylogenetic approaches. History in proteomes and careful biochemical annotation reveal that protein structural domains unfold in close interaction with cofactors, nucleic acids and membranes. This is unsurprising. Prebiotic chemistry dictates the emergence of numerous and efficient chemistries out of those that are possible. These chemistries are needed for later establishment of both a growing network of molecular interactions and new reactions catalyzed in pockets of emergent polymers. However and remarkably, phylogenomic analysis reveals that interactions of polypeptides with nucleic acids materialized for the first time later than interactions with cofactors and membranes, suggesting that genetics and its associated molecular functions were late additions to the functional repertoire of primordial life. The fact that genetic information was not a ‘first’ evolutionary development liberates us from explaining how nucleic acid polymers encoding molecular functions unfolded in the absence of their driver, the functional polypeptide chain, and how genetic information transformed into encoded information via the ribosome in that process [36]. However, the new model now forces us to explain (i) how memory was retained in the absence of modern genetics (necessary to fulfill the three axioms of evolution), and (ii) how genetic memory gradually unfolded to incorporate the universal translator, the ribosome. Here, memory refers to ‘instructions about the biological self’, i.e. self-referential information about the system that is passed in time with modifications imparted by interactions between the environment and the system itself.

The actual phylogenetic statements of proteome evolution can help gather explanations. Historical statements are tree-like relationships of phylogenetic taxa, the elements of structural accretion. Trees of protein structural domains describe the evolution of those domains and not the evolution of prior structural forms that are simpler and smaller in length. However, modern substructures must also fulfill the principle of continuity, i.e. they must depict and remember prior forms that existed at the beginning of life. Thus, mapping of prior forms to domains, such as mapping of dipeptides [59] or elementary functional loops of 25–30 residues in length [135] to structural domains, can give us insights into primordial recruitment processes responsible for modern structures and functions. In [59], we developed a phylogenomic-based model for the origin of modern genetics that starts with the development of structural *primordia* of catalytic domains of aaRSs acting as primordial dipeptidases and ligases and ending with the vectorial transfer of protein and nucleic acid structures to an emerging ribosome [57]. The emerging enzymatic activities were proposed to have biased the dipeptide sequence make-up of prior forms of domains, explaining considerable heterogeneities in the mapping of dipeptides to domains in genomes [59]. These compositional biases were likely driven by enhancements of the persistence of emerging cells, the absence of cellular lineages and the more-or-less free exchange of cellular components (e.g. by membrane ruptures, fissions and fusions) [57]. These drivers ensured the gradual build up of innovations that would benefit the entire cellular community. Biases also enhanced pre-existent amino acid biases poised by the likely abiotic synthesis of dipeptides and polypeptides, which we have not discussed. Phylogenomic analyses suggest initial enzymatic activities of molecular ancestors of aaRSs involved the ability to acylate a wide variety of cofactors (49-phosphopantetheine, CoA, NADP, and related derivatives, and short polynucleotides) in two-step catalytic reactions involving activated intermediates. These molecular functions are still embedded in the biochemical activities of aaRS enzymes [57]. Their structures had the potential to serve as primordial ligases of nucleotides and amino acid components. Their replicase functions were promiscuous. The outcomes were ‘fuzzy’ biochemistries with peptides and emerging proteins harboring quasi-statistical properties and manifesting only Rossmanoid and bundle folded structures, constrained by primitive membranes. Such structures were founders of the most basal fold structures of the phylogenomic timelines and the barrel structures of translation, including primordial structures of ribosomal proteins [56], [57]. In absence of modern genetics, any improvements of conformational stability and molecular activities that provided additional stability to primordial cellular envelopes and protein–nucleic acid interactions would have been fostered and later used to model improved genetic memory. Thus, genetics developed later than interaction with cofactors, membranes and metabolic pathways, but gradually captured prior improvements in the form of compositional memories.

In the model, history in RNA structure is also remembered and recovered with high explanatory power by the phylogenetic model of nucleic acid evolution. Recovery is even roughly congruent with nomothetic models once the core–periphery assumption of ‘rooting’ is removed (Fig. 7). The ribosome carries functions that are ‘processive’, i.e. functions that uniquely associate mechanical and biosynthetic molecular processes. It now appears that both of these functional aspects of the ensemble were recruited separately. Structures supporting decoding and ribosomal mechanics appeared first. They were perhaps hijacked from primitive replication machinery since the most ancient helical segments appearing before the ‘first evolutionary transition’ showed sequence and structure homologies with in vitro evolved RNA ligases and replicases [58]. These primordial RNA activities were likely associated to protein domain structures involved in the vectorial transfer between primordial aaRS-factor complexes and ribosomal components [57]. The biosynthetic core appeared at a later time, once the two major ribosomal subunits interacted fully to form the functional ‘turnstile’ that links decoding with biosynthesis. This ‘first evolutionary transition’ brings together tRNA interactions crucial to movement, catalysis, and intermolecular bridges necessary for the decoding-biosynthetic coupling. Thus, ribosomal history shows genetics preceded classic encoding and unfolded very quickly through functional cooption during the first transition identified in the timelines of ribosomal history.

## 8 The emergence of the ribosome and complex computation

What were the evolutionary drivers for ribosomal structure and function that would enable a nucleic acid code of protein structure? The biphasic model of module generation [13] we previously described can explain both the molecular aspect of the driver within the communal collective of primordial cells and the existence of a crucial ‘transition’ [59]. What about other more deeply entrenched drivers? We start with information.

Alan Turing proposed a theoretical machine that could compute any computable function [136]. In principle, his *Turing machine* was universal and could be used to construct any other computing machine. Indeed, John von Neumann used Turing's ideas to build a ‘universal constructor’, an automaton capable of self-replication and universal computation [137]. A standard Turing machine is a ‘program’ (an algorithm in the form of a *finite state machine*) controlling a mobile read-write head, which operates on an infinite tape (Fig. 4B). The tape is made of a string of placeholders for possible symbols given ‘states’ of the machine, which the head subjects to three operations: (1) read symbol in the tape's placeholder, (2) write (or overwrite) symbol, (3) move head (right or left) to adjacent symbol placeholders of the tape. Generally, when the machine starts operation the tape is blank except for some finite number of placeholders. However, the tape can be infinitely extended. Importantly, the ability of the head to edit the tape acts as memory of the computation. Three functions describe the Turing machine, *f_F_*, *f_G_* and *f_D_*, using Richard Feynman's nomenclature (Fig. 4B), the first two defining the current state of the finite state machine (*Q*) and its input symbol (*S*), and the third the direction of the head's displacement (*D*). Thus, a set of quintuples (*Q_t_*, *S_t_*, *Q*_*t* + 1_, *S*_*t* + 1_, *D*_*t* + 1_) defines what the machine will do for each symbol and state at time *t* + 1. A giant look-up of quintuples can be used to construct a ‘state table’, which defines the behavior of the machine for every possible combination of symbols and states.

A single universal Turing machine (*U*) can read tapes with descriptions of other Turing machines (*T*) and can therefore compute what these other machines can do. *U* consists of a finite state machine program controlling a mobile head operating on a tape. The tape contains data that completely describes machines *T*, including their data, program and functions. *U* can be constructed in different ways (Turing equivalents), including the concomitant use of a tape for storage of data about machines and a tape encoding the program. Such architecture has been successfully used to implement a Turing machine computer of field-programmable gate-array cells (digital boards) capable of self-replication and self-repair [138]. These theoretical constructs for computation, which are used to benchmark modern stored-program computer systems, can interpret the ribosome and its abilities of complex computation. A two-tape Turing machine resembles a ribosome, with one tape storing the program (RNA) and the other storing the data of machines (polypeptide). However, the heads of both tapes only move in one direction and do not have molecular editing capabilities. Consequently and deceivingly, the ribosome appears to be simply a finite state machine (a double turnstile), with all of its computational limitations. In fact, the inability of the natural ribosomal machine to erase symbols has been made evident in the recent construction of an artificial ribosome-like mechanical Turing machine for synthetic biology [139]. However, ribosomes are not isolated machinery but tightly integrated cellular components. It has been recently argued that Turing machines and cells have much in common [140]. Here we posit that ribosomal finite state machines gain editing functions *f_D_*(*Q*,*S*) and behave as *U* machines when cells of diversified and well-defined cellular lineages are selected for better performance. In this process, ‘data’ in the structure and function of polypeptides inform about cellular fitness. Cellular persistence poised by mutation then selects cells that harbor appropriate nucleic acid ‘programs’. These corresponding tunings at cellular level (cellular read–write heads) act as editing mechanisms for placeholders of the data and program tapes, turning ribosomal finite state machines (with read tapes) into *U* Turing machines (Fig. 4C). We note the importance of code discrimination embedded in tRNA, aaRSs and factors, which determine the symbols and states of the machines. Molecular discrimination is needed to build reliable computer programs that are resilient and avoid error.

The physical and functional disassociation between the ribosomal turnstile and the editing mechanism of diversified cells to fulfill biological ‘Turing computable’ operations in modern ribosomes has an important historical consequence. The turnstile finite state machine must develop earlier than a system of diversified cellular lineages that completed the natural *U* machine and enabled the rise of modern genetics. Since the *U* machine could have not originated before the last universal ancestor of cellular life, the editing properties of emerging lineages would have not been remembered and genetics could have not arisen before that time. In contrast, the memory for its turnstile could have unfolded without genetics and prior to diversified cells, as our phylogenomic explorations reveal. More importantly, the rise of complex ribosomal computation can now be regarded as a likely driver of cellular diversification, starting with molecular finite state machines and ending with the universal computational capabilities of modern cells and organisms.

## 9 Summary and outlook

There is a functional logic to the accretion process in molecules. The ribosomal ‘turnstile’ for example has moving parts that need to be located in the periphery as the multimolecular complex grows, regardless of the early or late origin of its moving and fix components. This imposes inward growth tendencies that push some translocation structures outward. In turn, the central ratchet and PTC center are central mechanisms and catalytic centers that benefit from gradual stabilization induced by external layers of apical growth. Despite this functional rationale, the mappings of ages of ribosomal components onto the molecules show an evolutionary patchwork (Fig. 5, Fig. 7B) similar to patchworks observed in metabolic networks (Fig. 3). The patchwork mode appears to override the gradual ‘layering’ mode responsible for core–periphery patterns in molecular repertoires and molecules. In fact, the ideographic methods reveal patchwork patterns of ribosomal accretion that contradict the concentric layering inferred using nomothetic approaches under the premise of outward growth. Coaxial helical stacking distributions in structure now suggest that genetic insertions cause growth scenarios that are different, involving helix reformations and late establishment of helical stacking and other tertiary interactions. These alternative processes make insertion fingerprints deceiving. They lessen the primacy of the gradual mode of evolution. Peripheral points of origin, such as structures supporting the PTC and the L1 stalk of domain V, demand growth by small or large tandem duplications. The H73 helix that connects the PTC to the central junction and the base of the molecule must be basipetally constructed inward to close the subtending 5-way junction. The H74 and H75 stems must be extended inward in growing ribosomes to preserve the peripheral translocation functionalities of the subtending junction [112]. This is necessary because these regions are distal to the base of the molecule and their functionality crucial. But, what if primordial RNA molecules were many and shorter? There would be less distance to traverse. Indeed, unusual functional ribosomes exist in basal eukaryotes that are made up of covalently non-continuous rRNA (e.g., [141]). Thus, primordial ribosomes could have been composed of separate interacting pieces, each contributing specific functions (see [142] for a historical account). The fragments would have joined in evolution in most lineages, leaving behind only the deceiving inward growth patterns. A recent proposal posits that the ribosome is a vestige of an ancestral genome composed of multiple primordial tRNA [143]. If the proposal is correct, the ribosome and genome must be linked by imprints of primordial complementarity similar to those uncovered in tRNA [144]. In fact, even tRNA molecules could have been produced from pieces [145]. Thus, hypotheses of structural grafting of multiple growing rRNA molecules help explain the phylogenomic patchwork uncovered by ideographic analysis, and could reconcile molecular history and process evolution.

Coaxial helical stacking patterns also prompt careful integration of phylogenetic and structure-based evolutionary models with molecular biology bench work and biophysics to address ribosomal growth and evolutionary constraints acting on ribosomal structure. Yokohama and Suzuki [146] recently explored the functional capability of rRNA by systematically inserting 32 nucleotide-long segments into *E. coli* rRNA. They found that most insertions coincided with eukaryotic expansion segments. Most of them were located in peripheral regions, but some of them were close to the 10-way central junction of the molecule that is close to its base. These results show ribosomal structure is malleable and remarkably tolerant to change. Since insertions follow patterns of apical growth, it is therefore likely that processes driving accretion of recent ribosomal layers may be different than those responsible for the ancient ribosomal core. We note that the size of the folded rRNA molecules and domains measured with the radius of gyration follows the Flory scaling law and the shape computed using the eigenvalues of the moment of inertia tensor shows they are considerably aspherical, preponderantly prolate and flexible (especially domains II, IV, V and VI) and loosely packed (compared to proteins)[147]. The entire ribosome and large subunit, however, are both quite globular. These features are probably acquired after folding and result from a larger number of small helices and a relatively low number of coaxial stacking interactions in folded LSU rRNA. The shape of intact ribosomes and its constituent parts suggest that folding of the individual components might occur prior to assembly, a feature that holds the hallmark of modularity. A model of layered evolution is therefore unlikely, since it would be incompatible with the biophysics of ribosomal components.

A number of questions now arise from patterns of macromolecular accretion. Are they the result of functional recruitment? Are recruited pieces modules or the products of stochastic processes of genetic insertions and rearrangements? Can they be fully explained by a biphasic model of module generation? Does accretion arise from processes that are heterogeneous in time and space? Are primordial and modern accretion processes different? These questions are important. The recognition of a possible frustrated dynamics of molecular growth is necessary for synthetic biology. Biological engineering must take into consideration how biological molecules unfold so that they can be appropriately designed for medical and industrial applications.

## Figures and Tables

**Table 1:** Glossary of selected terminology.

Term	Definition
Character	In phylogenetics, a character is an observable feature of a biological entity (primary homology) that is used to establish its history. Characters have alternative manifestations (character states) and are most powerful when they unfold as ‘shared and derived’ features (synapomorphies) in evolutionary tree or network hypotheses.
Character transformation	A series of character states that transform into each other in evolution.
Character polarization	Assignment of polarity to a character transformation. Polarity implies specifying direction of character change and which states are evolutionarily ancestral and which are derived. Character polarization enables the rooting of phylogenetic trees or networks and the identification of synapomorphies.
Dynamical system	A natural object delimited by a set of interacting component subsystems, which is characterized and individuated from other systems by its cohesion [11]. Cohesion refers to the dynamical stabilities of the components of the system, its parts, when these are constrained by the system as a whole. Consequently, systems are by definition decomposable into subsystems that are either spatially bounded (e.g. nucleotides in nucleic acids) or spatially unlinked (e.g. processes or other dynamical entities). Cohesion is the property of modules, a special group of parts.
Hennigian argumentation	An explicit procedure of retrodiction that uses character transformations (evolutionary models) and phylogenetic information in individual characters (data) to build tree or network hypotheses of evolution
Hennigian (reciprocal or mutual) illumination	A successive approximation route for developing scientific theories of evolution, in which additional evidence in the form of more informative phylogenetic characters is added to a corpus of ideographic evidence to support the validity of phylogenetic hypotheses.
Finite state machine	A mathematical model of computation conceived as an abstract machine (black box) that can be in one of a finite number of states, one state at a time. A state can transition into another state induced by a stimulus or input. A typical example is a coin-operated turnstile with two-states (locked and unlocked) and two inputs (insert coin and push turnstile mechanical arm). See Fig. 4.
Frustrated dynamics	Patterns of change and behaviors of systems governed by competing and often opposing forces. Examples of frustration include spin glasses, which are important for condensed matter physics. In biology, RNA folding for example follows a frustrated dynamics, in which structural conformations that are formed are both stabilized by hydrogen bonding interactions between bases and destabilized by unpaired regions of the molecules.
Laplacian demon	Pierre Simon Laplace in his *Essai philosophique sur les probabilities* (1814) articulated the rationale of causal or scientific determinism: “We may regard the present state of the universe as the effect of its past and the cause of its future. An intellect which at a certain moment would know all forces that set nature in motion, and all positions of all items of which nature is composed, if this intellect were also vast enough to submit these data to analysis, it would embrace in a single formula the movements of the greatest bodies of the universe and those of the tiniest atom; for such an intellect nothing would be uncertain and the future just like the past would be present before its eyes.” His demon represents a utopian super-intelligence capable of perfect foreknowledge.
Lundberg rooting	A method of rooting that first determines an optimal tree or network and then adds a hypothetical ancestor (defined by all-ancestral state characters) at the position in the tree or network that is most optimal.
Matrix transposition	Mathematical operation in which a matrix is converted into a new matrix whose rows are the columns of the original.
Node	A point in a phylogenetic tree or network where three or more branches meet.
Phylogeny	A hypothesis of genealogical relationships among a group of entities (taxa) in the form of a tree or network with specific connotations of ancestry and an implied time axis.
Ribosomal translocation	Movement of the codon-anticodon duplices on SSU from A and P sites to the P and E sites, respectively [112].
Weston's generality criterion	A general criterion of character polarization capable of distinguishing ancestral and derived character states. The criterion, inspired by Nelson's ontogenetic rule, states: “Given a distribution of two homologous characters in which one, x, is possessed by all of the species [taxa] that possess its homolog character y, and by at least one other species that does not, then y may be postulated to be apomorphous [derived] relative to x.” It is based on homology and additive phylogenetic change, and is most powerful when features of characters accumulate ‘iteratively’ in evolution (e.g. gene paralogs via duplication).

**Table 2:** Putative insertions in LSU rRNA.

Insertion name and type		Linked AES	Trunk helices	Trunk ages (nd)	Branch helices	Branch ages (nd)	C^a^	JO^b^	Model	V^c^
B1	Basipetal	41,53	H8, H9	0.89, 0.72	H5, H10	0.87, 0.65	−	5	Branches older than trunk	−
B2	Basipetal	9,10	H35, H34	0.94, 0.54	H33	0.41	−	3	Branch older than trunk	−
B3	Basipetal	14,16	H37, H38	0.44–0.04	H36, H39	0.69–0.37	−	7	Branch older than trunk	−
B4	Basipetal	22,23	H41, H45	0.04–0.41	H36, H40	0.69–0.91	−	7	Trunk older than branches	+
B5	Basipetal	9,14	H36, H46	0.69–0.57	H26, H32	0.56–0.41	−	5	Branches older than trunk	−
B6	Basipetal	35,47	H53, H54	0.70, 0.54	H51, H52	0.91, 0.65	−	4	Branch older than trunk	−
B7	Basipetal	9,28	H47, H48	0.67, 0.94	H26a	1	−	4	Trunk older than branch	+
B8	Basipetal	8,9	H26, H26a	0.56–1.00	H25a, H72	0.18–0.69	−	10	Branches older than trunk	−
B9	Basipetal	1,3	H76, H79	0.04–0.67	H75	0.3	−	3	Branch older than trunk	−
B10	Basipetal	36,40	H86, H87	0.54, 0.87	H82, H83	0.96, 0.48	−	4	Branch older than trunk	−
B11	Basipetal	1,39	H74, H89	0.30–0.30	H73, H90	0.30–0.30	−	5	Same age	?
B12	Basipetal	7,30	H95, H96	0.61, 0.13	H94, H97	0.28, 0.59	−	4	Branches older than trunk	−
B13	Basipetal	7,50	H98, H99	nd, 0.72	H1, H94	nd, 0.29	−	10	Branch older than trunk	−
A1	Apical	41,59	H5, H6	0.87, 0.82	H7	0.39	+	3	Branch older than trunk	−
A2	Apical	21,41	H5, H10	0.87, 0.69	H4, H8, H9	0.37, 0.89, 0.72	+	5	Branch older than trunk	−
A3	Apical	52,55	H18, H20	0.89, 0.56	H19	0.56	+	3	Same age	?
A4	Apical	9,13	H31, H32	0.57, 0.41	35a	0.61	+	3	Trunk older than branch	+
A5	Apical	32a,38	H42, H44	0.63, 0.04	H43	0.63	+	3	Same age	?
A6	Apical	51,56	H56, H57	0.59, 0.35	H58, H59	0.67, 0.98	+	4	Trunk older than branch	+
A7	Apical	31,35	H50, H51	0.52, 0.91	H49, H49a	0.70, 0.91	+	4	Branch older than trunk	−
A8	Apical	11,12	H64, H67	0.74, 0.07	H65, H66	0.37, 0.76	+	4	Branch older than trunk	−
A9	Apical	2,27	H80, H88	0.70, 0.43	H74, H75, H81, H82	0.30, 0.30, 0.67, 0.96	+	6	Branch older than trunk	−
A10	Apical	1,2	H74, H75	0.30, 0.30	H81, H82, H80, H88	0.67, 0.96, 0.70, 0.43	+	6	Trunk older than branch	+
A11	Apical	3,5	H90, H91	0.3, 0.43	H92	0.72	+	3	Trunk older than branch	+
A12	Apical	7,30	H94, H97	0.28, 0.59	H95, H96	0.61, 0.13	+	4	Branch older than trunk	−

a C, Compatible with acropetal insertion model of molecular growth (−, no; +, yes).

b JO, Junction order.

c V, phylogenetically-validated insertion (−, no; +, yes; ?, inconclusive).
